# Molecular docking and dynamics simulation studies uncover the host–pathogen protein–protein interactions in soybean (*Glycine max* (L.) Merr.) and *Groundnut bud necrosis virus*: first report

**DOI:** 10.3389/fbinf.2026.1821527

**Published:** 2026-07-31

**Authors:** Rayees Ahmad Bhat, Ayyagari Ramlal, Amooru Harika, Ashutosh Sharma, Ambika Rajendran, Iten M. Fawzy, Sreeramanan Subramaniam, Dhandapani Raju, S. K. Lal, Sonu Krishankumar, Shyam S. Kurup

**Affiliations:** 1 Department of Botany, University of Delhi, Delhi, India; 2 School of Biological Sciences, Universiti Sains Malaysia (USM), Georgetown, Penang, Malaysia; 3 Division of Genetics, ICAR-Indian Agricultural Research Institute (IARI), New Delhi, New Delhi, India; 4 Plant Sciences, Department of Plant and Environmental Sciences, Clemson University, Clemson, SC, United States; 5 RNA Biology Laboratory, Department of Chemistry, Indian Institute of Technology, Hauz Khas, New Delhi, India; 6 Department of Pharmaceutical Chemistry, Faculty of Pharmacy, Future University in Egypt, Cairo, Egypt; 7 Centre for Chemical Biology (CCB), Universiti Sains Malaysia (USM), Bayan Lepas, Penang, Malaysia; 8 Department of Biology, Faculty of Science and Technology, Universitas Airlangga, Surabaya, Indonesia; 9 Agro-Biotechnology Institute, National Institute of Biotechnology Malaysia (NIBM), Selangor, Serdang, Malaysia; 10 Division of Plant Physiology, ICAR-Indian Agriculture Research Institute (IARI), New Delhi, New Delhi, India; 11 Integrative Agriculture Department, College of Agriculture and Veterinary Medicine, United Arab Emirates University (UAEU), Al Ain, United Arab Emirates; 12 Khalifa Centre for Genetic Engineering and Biotechnology, United Arab Emirates University (UAEU), Al Ain, United Arab Emirates

**Keywords:** biotic stress, *Groundnut bud necrosis virus*, herbal remedies, *in silico* prediction, soybean, tolerance

## Abstract

**Background:**

Soybean is a widely consumed oilseed crop with economic importance. It is enriched with numerous bioactive compounds that have health-promoting properties. *Groundnut bud necrosis virus* (GBNV) affects many agriculturally important crops, such as soybean, resulting in crop losses. GBNV is a single-stranded RNA virus from the genus Orthotospovirus and family Bunyaviridae. It is transmitted by insect vectors (thrips) and is further aggravated by secondary transmission from infected plants to others in the same field. There are no commercially available drugs against GBNV; thus, there is a need to explore phytomolecules that control the infection. This study utilizes soybean proteins (CAM, CDK1, Cul1, GSK3, HSP70, LOX, PCNA, and TCP) and GBNV proteins (CP, EGP, MP, NSP, NS, and RDRP) for the analysis of their inhibitory interactions. Physicochemical properties for both protein groups were examined. The molecular basis for the selective predictive association of these protein interactions was evaluated using *in silico* molecular docking approaches, dynamics simulations analysis, and post-simulation computational analyses.

**Results:**

The results indicate that among these 50 protein–protein interactions, heat shock protein 70 (HSP70) exhibited potential inhibitory action against the RNA-dependent RNA polymerase (RDRP) with −12.12 kcal/mol binding affinity (z-score: 0.0). RDRP is an essential enzyme required for the transcription and replication of the viral genome. HSPs function in both abiotic and biotic stresses. Although further studies are required, these preliminary findings will be useful for developing new therapeutic agents against the virus, thus paving the way for researchers to find better alternatives.

**Conclusion:**

This is the first study to combine prediction, structural validation, and interface analysis of the interaction between soybean and GBNV proteins.

## Introduction

Soybean (*Glycine max* (L.) Merr.) is an important multipurpose food and oilseed leguminous crop. It contains approximately 40% protein and approximately 18%–20% oil (Sreenivasulu et al., 2008; Modgil et al., 2021). It is also a source for animals as a feed crop (Medic et al., 2014). The global importance of soybeans is apparent in the fact that they contribute to the production of approximately 25% of edible oils globally, constitute 57% of the world’s seed-oil production, and also contribute to two-thirds of the global protein feed for livestock (Pratap et al., 2012; Hill and Whitham, 2014; Maranna et al., 2021). Soybean contains various bioactive components, including saponins, polysterols, flavonoids, lecithins, and other compounds (Modgil et al., 2021). Previous studies on the angiotensin-converting enzyme showed that soybean proteins and phytocompounds can lower hypertension and reduce the risk of cardiovascular diseases (Ramlal et al., 2022a; Ramlal et al., 2022b; Ramlal et al., 2023a; Ramlal et al., 2024a). Similarly, soy compounds are effective against the malaria-causing bacterium *Plasmodium falciparum* (Ramlal et al., 2024b).

Plant viruses infect different crop species, causing economic losses and, thereby, leading to threats to food security (Haňcinský et al., 2020; Ramlal et al., 2023b; Ramlal et al., 2023c). Among these viruses Tospoviruses have gained much attention due to their widespread distribution and devastating effects on a wide range of economically important crops (vegetables, legumes, and ornamentals). *Groundnut bud necrosis virus* (GBNV) also belongs to the Orthotospovirus genus, family Bunyaviridae. GBNV has a quasispherical (80 nm–120 nm diameter) envelope containing a negative single-stranded RNA (ssRNA) genome having a tripartite genome containing small (S), medium (M), and large (L) segments of ssRNA. The S RNA contains 3.0 kb, M RNA has 5.0 kb, and L RNA has 9.0 kb of genome size (Reddy et al., 1995; Rai et al., 2020; De Castro et al., 2024; Deraniyagala et al., 2025; Jamwal et al., 2025). In the early 1990s, GBNV was identified as the first distinct Orthotospovirus species that caused the disease in groundnuts in India (Reddy et al., 1991).

The L RNA encodes the polymerase. The M segment, having an ambisense orientation, produces nonstructural movement (NSM) protein in the sense direction. The NSM is a precursor to the membrane glycoproteins Gn and Gc in a complementary sense. The sense nonstructural (NSS) protein is encoded by the S RNA and the complementary sense nucleocapsid (NP) protein (Satyanarayana et al., 1996; Gowda et al., 1998; Akram et al., 2004; Mandal et al., 2012; Deraniyagala et al., 2025). Economically, the most significant Orthotospovirus in India is GBNV, that affects soybeans. Like other Orthotospoviruses, GBNV is also transmitted by several thrips species, including *Thrips palmi* and *Scirtothrips dorsalis,* leading to characteristic symptoms such as bud necrosis, chlorotic rings, and stunted plant growth. Under natural conditions, the main characteristic symptom is necrosis of the terminal bud (Lakshmi et al., 1995; Meena et al., 2005; Basavaraj et al., 2017; Jamwal et al., 2025). The disease affects the yield of soybean significantly, making it a concern for farmers and researchers. Viruses, including GBNV, can cause different diseases in soybeans, which continue to affect the yield and result in significant losses to producers. As soybean is one of the crucial sources of food, protein, and oil, further research is necessary to enhance their yield under various environmental stresses (Bhat et al., 2002; Pagano and Miransari, 2016).

This study aims to utilize various *in silico* approaches to analyze soybean and GBNV proteins, sequence analysis, predict their structural features, and investigate potential host–virus interactions using the selected proteins through *in silico* molecular docking approaches. These interactions will be utilized to identify key viral targets that can facilitate the development of resistant soybean varieties and effective antiviral strategies.

## Materials and methods

**FIGURE 1:**
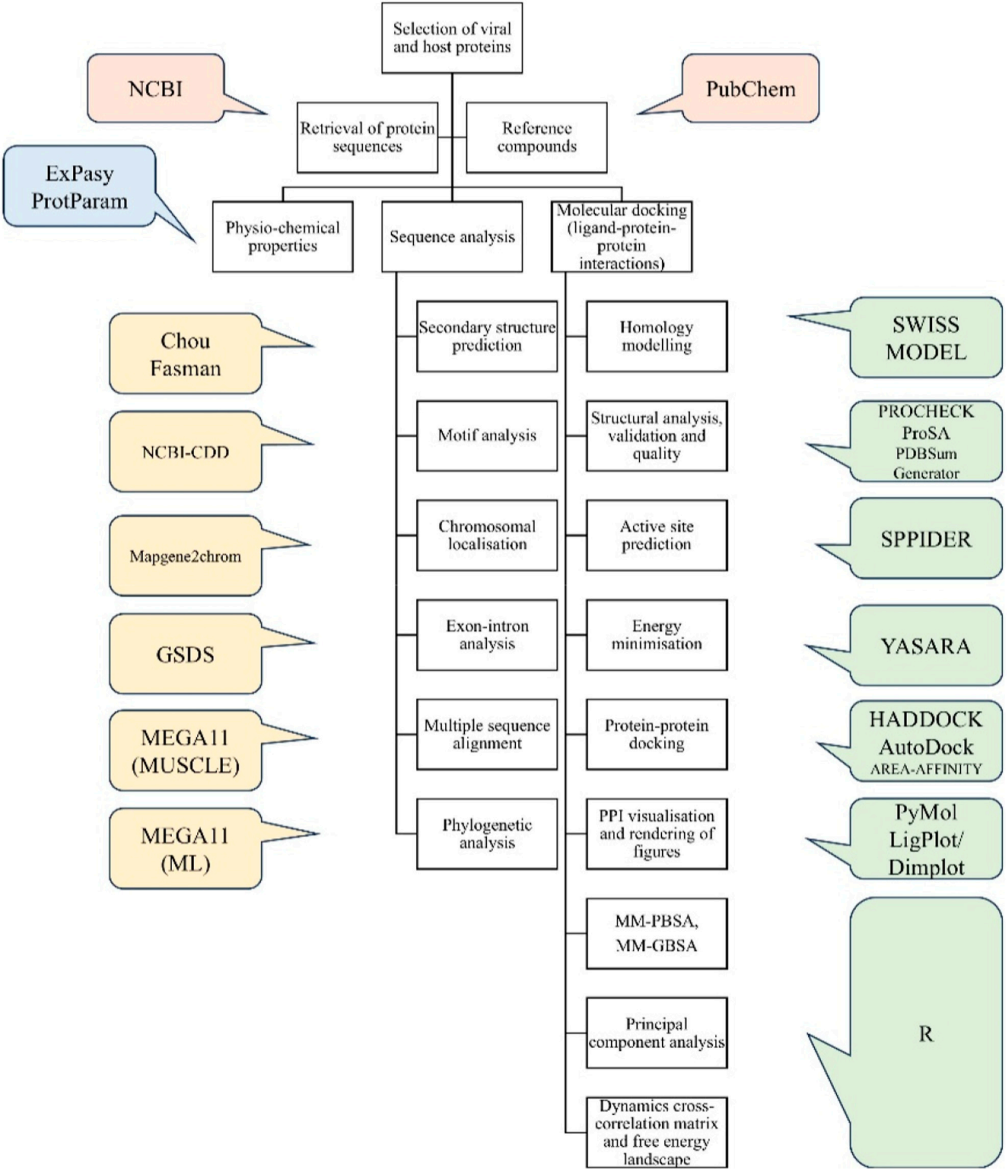
Pictorial representation of the workflow of the study.

The workflow of the study is shown in Figure 1.

### Sequence analyses: sequence and structural features

#### Sequence retrieval

GBMV proteins, namely, CPGBNV (NCBI: XDU23736), NSGBNV (NCBI: AWV56699), NSPGBNV (NCBI: AWV56697), EGPGBNV (NCBI: AWV56696), RDRPGBNV (NCBI: AWV56695), and MPGBNV (NCBI: UVD61612), and soybean proteins including PCNA (NCBI: NP_001241553), CDK1 (NCBI: KAH1220609), CaM (NCBI: NP_001237902), GSK3 (NCBI: NP_001242661), HSP70 (NCBI: NP_001362769), Cul1 (NCBI: XP_006604710), TCP (NCBI: AGP03826), and LOX1 (NCBI: AAA96817) were retrieved from the NCBI (https://www.ncbi.nlm.nih.gov/). Two reference compounds: ganoderic acid (GAA) (PubChem: 471002) and squalene (Sq) (PubChem: 638072) were retrieved from PubChem (https://pubchem.ncbi.nlm.nih.gov/) (Supplementary Figure S1).

#### Physicochemical properties, subcellular localization, transmembrane analysis, and secondary structure prediction

*Physiochemical parameters*: The molecular weight, isoelectric point (pI), instability index (II), aliphatic index (AI), and grand average of hydropathicity (GRAVY) were computed using ProtParam (https://web.expasy.org/protparam/) (Gasteiger et al., 2005). *Subcellular localization*: Subcellular localization was assessed using the PSORT (https://psort.org/) tools (Nakai, 2000; Emanuelsson, 2002), including iPSORT (https://ipsort.hgc.jp/) (Bannai et al., 2002) and WoLF PSORT (https://wolfpsort.hgc.jp/) (Horton et al., 2007). *Transmembrane helices (TMH)*: The presence of TMH was computed using https://services.healthtech.dtu.dk/service.php?TMHMM-2.0. *N-terminal*: The presence of signal peptide (SP), mitochondrial (mt), or chloroplast (c) signal sequence was assessed using https://services.healthtech.dtu.dk/service.php?TargetP-2.0. *Prediction of serine (S), threonine (T), and tyrosine (Y)*: These were predicted using the NetPhos 3.1 (https://services.healthtech.dtu.dk/services/NetPhos-3.1/, Blom et al., 1999; Blom et al., 2004). *Secondary structure prediction*: The secondary structures were computed using the Chou and Fasman secondary structure prediction server (http://www.biogem.org/tool/chou-fasman/) (Kumar, 2013). The candidate proteins were confirmed for the presence of the specific protein family by HMMER software (http://hmmer.org/) with the Pfam motif IDs and simple modular architecture research tool (SMART; https://smart.embl-heidelberg.de/) (Letunic and Bork, 2018; Letunic et al., 2021).

#### Hydrophobicity plot

The hydrophobicity of actin proteins was analyzed using BioEdit 7.2.5, with a window size of 13, based on the Kyte–Doolittle scale mean hydrophobicity profile (Kyte and Doolittle, 1982; Hall, 1999; Li et al., 2019; Ramlal and Samanta, 2022).

#### Exon–intron structure and chromosomal localization

The exon–intron structure was analyzed using the gene structure display server (GSDS; https://gsds.gao-lab.org/) (Hu et al., 2015). The chromosomal location of the host genes is based on the genome annotation information provided in the NCBI database and drafted by the MapGene2Chrom online tool (http://mg2c.iask.in/mg2c_v2.0/(Chao et al., 2021).

#### Calmodulin-binding site prediction

The calmodulation database predicted putative calmodulin-binding site motifs and a meta-analysis predictor (https://cam.umassmed.edu/index.php) and the calmodulin target database (http://calcium.uhnres.utoronto.ca/ctdb/pub_pages/search/index.htm) for viral and host proteins (Yap et al., 2000; Mruk et al., 2014).

#### Motif and conserved domain analyses

Multiple expectation maximization for motif elicitation (MEME) was used to identify conserved motif structures among protein sequences (https://meme-suite.org/meme/tools/meme) (Bailey and Elkan, 1994). The protein domain regions or functional regions were identified using the conserved domain database (CDD), and their putative roles were searched using CDD-NCBI (https://www.ncbi.nlm.nih.gov/Structure/cdd/wrpsb.cgi) (Marchler-Bauer et al., 2017; Wang et al., 2023).

#### Multiple sequence alignment and phylogenetic analyses

A WebLogo was created for the conserved patterns of amino acids with respect to their positions in the host and viral proteins using multiple sequence alignment (MSA) with MEGA12 (V: 12.0.11). The sequence logo was generated by using WebLogo 2.8.2 (https://weblogo.berkeley.edu/logo.cgi) (Crooks et al., 2004).

The MSA of both GBNV and soy proteins was carried out using MEGA11 (V: 11.0.13) and MEGA12 (Tamura et al., 2004; Tamura et al., 2021; Kumar et al., 2024; https://www.megasoftware.net/) with the MUSCLE algorithm using default parameters. The obtained MSA was used for the construction of phylogenetic trees for the gene, CDS, and protein sequences of both GBNV and soy using the neighbor-joining (NJ) method with 1,000 bootstraps (Gascuel, 1997; Felsenstein, 1985; Saitou and Nei, 1987; Jones et al., 1992).

#### Structure prediction through homology modeling

Host and viral proteins models were built using both the SWISS-MODEL workstation (https://swissmodel.expasy.org/interactive) (Waterhouse et al., 2018) based on the values of QMEAND (Waterhouse et al., 2018; Studer et al., 2020) and QMEAN (Benkert et al., 2011) and the Phyre2.2 Protein homology/analogy recognition engine (V: 2.2, Phyre2; https://www.sbg.bio.ic.ac.uk/phyre2/html/page.cgi?id=index) using the normal mode based on the maximum confidence and coverage scores (Powell et al., 2025).

#### Model validation of viral and host proteins and Ramachandran plot

The viral and host protein models were subjected to structural validation and quality using the PDBsum Generate server (https://www.ebi.ac.uk/thornton-srv/databases/pdbsum/Generate.html). The energy potential of the predicted models was calculated using the protein structure analysis (ProSA) (https://prosa.services.came.sbg.ac.at/prosa.php) (Wiederstein and Sippl, 2007), and SAVES (V: 6.1; https://saves.mbi.ucla.edu/) for the Verify3D (Bowie et al., 1991; Lüthy et al., 1992) and PROCHECK (Laskowski et al., 1993) scores were used to verify the quality of the predicted models. ERRAT (Colovos and Yeates, 1993) was performed to estimate the accuracy of the non-bound atoms of the modeled proteins. Furthermore, the Ramachandran (RC) plots for the native forms of the proteins were constructed using RamPlot (https://www.ramplot.in/) and PyMol (V: 2.5.4; https://www.pymol.org/) (DeLano, 2002; Yuan et al., 2017; Kumar and Rathore, 2025).

#### Energy minimization and active site prediction

The energy minimizations (EM) for both the proteins and ligands were carried out (http://www.yasara.org/minimizationserver.htm) (Krieger et al., 2009; Ozvoldik et al., 2023). Solvent accessibility-based protein–protein interface identification and recognition (SPPIDER; https://sppider.cchmc.org/) was utilized for the identification of active sites of the proteins (Porollo and Meller, 2007; Porollo and Meller, 2012).

### Molecular docking: protein–protein/ligand docking, dynamics, and post-MD computational analyses

#### Protein–protein docking

Protein–protein docking was performed using high-ambiguity-driven protein–protein docking HADDOCK (V: 2.5.2024.12; https://rascar.science.uu.nl/haddock2.4/) (Honorato et al., 2021; Honorato et al., 2024). The binding affinities were calculated using the “protein–protein” method from the area-affinity web-server (https://affinity.cuhk.edu.cn/) (Yang et al., 2022; Yang et al., 2023).

### Protein–ligand docking

#### Preparation

For the docking experiments, the ligands were prepared using Chimera (V: 1.15; https://www.cgl.ucsf.edu/chimera/download.html), and AutoDock Vina (V: 1.5.7) (http://vina.scripps.edu/download.html) was used for the preparation of the proteins. Heteroatoms and water were removed, polar hydrogens were added, nonpolar hydrogens were merged, and both Kollman and Gasteiger charges were added. Ligands downloaded from PubChem in the SDF format were converted into the Protein Data Bank (PDB) format using OpenBabel (http://openbabel.org/wiki/Main_Page). Charges of the ligands were set to neutral, and the Gasteiger charge was added. The number of torsions was kept as the default.

#### Docking

Docking was performed using the AutoDock Vina tool (Trott and Olson, 2010). Blind docking was performed initially, followed by precision docking. The spacing angstrom was set to 1, and then the grid box was adjusted manually to cover all the active site residues of the protein. The dimensions of the grid box were set as X = 30, Y = 30, and Z = 30, and the center grid box was set with the coordinates as center x = 150.309, center y = 119.844, and center z = 83.415 for RDRP and GAA, while for RDRP with Sq, the coordinates were center x = 156.105, center y = 120.646, and center z = 86.369, and the grid box dimensions were X = 30, Y = 30, and Z = 30. Exhaustiveness was set to 8 for both dockings. All the dockings were performed in three replicates (Morris et al., 2009; Cosconati et al., 2010; Forli and Olson, 2012).

#### Inhibition constant

The inhibition constant (Ki) is calculated from the obtained binding energy (ΔG) using Equation 1 (Ortiz et al., 2019).

$$K_{i}=e^{\frac{\Delta G}{RT}}$$ (1)

where R is the universal gas constant (1.985 × 10^−3^ kcal/mol K) and T is the temperature (298.15 K).

#### Absorption, distribution, metabolism, excretion, and toxicity prediction studies

The ligands (GAA and Sq) were subjected to absorption, distribution, metabolism, excretion, and toxicity (ADMET) studies using SwissADME (http://www.swissadme.ch/) (Daina et al., 2017) to determine and analyze the pharmacokinetic properties of the molecules.

#### Molecular dynamics

Molecular dynamic (MD) simulation studies were performed using Discovery Studio 4.0 software against the most suitable templates, which were PDB: 8KI7 for the RDRP and UniProt: A0A6P5U5G1 (heat shock protein-like of *Prunus avium* (L.) L. and was converted it into a 3D model structure) for the HSP70. Studies were conducted on the docked forms of the enzymes and compared to their free protein states. Standard dynamic cascades were applied, with the first minimization algorithm set to steepest descent, allowing for a maximum of 2,000 steps and a root mean square (RMS) gradient of 1.0. The second minimization algorithm was set to a conjugate gradient with 2,000 maximum steps, and the RMS gradient was equal to 0.1. Three phases were applied: heating, equilibration, and production for a time of 600 ns. The initial temperature of 50 and a target temperature of 300, with a maximum velocity of 2,000, were adjusted. For the implicit solvent model with the leapfrog Verlet dynamics integrator protocol, NVT and the generalized born with simple switching were adjusted (Ramlal et al., 2024b). The Z-Docker protocol was adopted to view the possible interactions between both proteins, and the resulting poses were refined according to the best Z-rank score of the poses in each produced cluster (Ramlal et al., 2024b).

#### Molecular mechanics/Poisson–Boltzmann surface area (MM/PBSA) and molecular mechanics/generalized born surface area (MM/GBSA) binding free energy calculation

The binding free energy of a system can provide a mechanistic insight into protein–ligand interactions. Therefore, MM/PBSA and MM/GBSA were also computed for the proteins (8KI7 and 4HDH) and ligands (GAA and Sq) (Singh et al., 2024; Pandi et al., 2026).

#### Dynamic cross-correlation matrix plot

The dynamic cross-correlation matrix (DCCM) analysis was carried out to evaluate the correlated and anti-correlated motions among amino acid residues throughout the MD simulation. The DCCM was generated from the C-alpha atom trajectories of the protein by using standard correlation algorithms. Positive correlation values indicated coordinated movement between residues, whereas negative values represented motion in the opposite direction (Arnold and Ornstein, 1997; Prada-Gracia et al., 2009; Shibu et al., 2026).

#### Principal component analysis and free energy landscape plot

The fluctuations in protein residues on PL binding were observed through principal component analysis (PCA), and the free energy landscape (FEL) analysis was performed to determine the conformational stability and energy minima of the PL complex using the MD (Prada-Gracia et al., 2009; Shibu et al., 2026).

#### Visualization of poses

PyMoL (https://www.pymol.org/pymol.html) is used for the visualization and analysis of the docked poses and protein structures and for rendering the figures, while LigPlot and Dimplot of LigPlot+ (V: 2.2.9) were used to obtain the two-dimensional PL and protein–protein interactions (PPIs), respectively (Wallace et al., 1995; Laskowski and Swindells, 2011; Schrodinger, 2021).

#### Data analysis

The PCA, DCCM, and FEL analyses were carried out using R V: 2026.01.2 + 418 (Supplementary Material).

## Results

### Physicochemical properties, subcellular localization, transmembrane analysis, and secondary structure prediction

The SMART tool was used to confirm that the candidate sequences contained the specific domains (Table 1). Following that, the physicochemical properties of all the identified sequences were computed (Table 2). For the viral proteins, the length of the amino acids (aa) ranged from 271 (coat protein; CP) to 2,878 (RNA-dependent RNA polymerase; RDRP). Similarly, the molecular weight (MW) ranged from 29.97 kDa for the coat protein to 331.31 kDa for RDRP. The proteins have shown differences in the isoelectric points, with 5.95 for the envelope glycoprotein, while that of the other proteins ranged from 6.89 (non-structural protein; NS) to 8.79 (CP) (Table 2). For the host proteins, the aa length varied from 103 to 839 for cyclin-dependent kinase regulatory subunit 1 (CDK1) and lipoxygenase (LOX1), respectively. The MW ranged from 12.36 (CDK1) to 96.3 kDa (LOX1). The PIs also varied from the lowest value of 4.04 for calmodulin (CaM) to 10.17 for TCP family transcription factor (TCP), while other values are mentioned in Table 2. The II represents the ability of a protein to remain stable in a test tube following its isolation. The II should be less than 40 (<40) for a protein to be considered stable; otherwise, the protein is considered unstable (Guruprasad et al., 1990). In this study, both the protein groups showed stable and unstable proteins. For instance, among viral proteins (VPs), the NS and EG were stable, while among the host proteins (HPs), the CaM, glycogen synthase kinase 3 (GSK3), heat shock 70-kDa protein (HSP70), and LOX1 were found to be stable. The AI shows the relative volume of aliphatic side chains occupied in a protein chain, which in turn can be correlated with the thermostability of a protein (Ikai, 1980). Here, the AI was found to range from 78.52 to 90.77 for VPs and 81.87 to 91.71, except 61.03, for TCP. The GRAVY represents the sum of hydropathy values (Kyte and Doolittle, 1982). The negative value shows that the proteins are hydrophilic in nature (whereas positive values show that they are hydrophobic), which is supported by the fact that most of the proteins reside in the cytoplasm (also shown in the *Hydropathicity* section below). All the VPs and HPs have negative values, except for the movement protein of the virus, which has a positive value of 0.371. Being viral proteins, the signal peptides, transmembrane regions, and the subcellular localization analyses were not performed. The subcellular localization of the sequences predicted shows that they can be found in the nucleus, cytoplasm, chloroplast, cytoskeleton, Golgi apparatus, plastids, mitochondria, and peroxisomes (Bhat et al., 2025). RDRP contains the highest number of serine (S), threonine (T), and tyrosine (Y) phosphorylation sites, namely, 176, 74, and 41, respectively, from the viral proteins, while in the host proteins, LOX1 contains the greatest number of S (43) and T (35) sites; however, Y sites are more abundant in Cul1 (14) (Table 2).

**TABLE 1:** SMART motif analysis of A) viral and B) host proteins.

(A)					
Protein	From	To	e-Value	Domain	InterPro ID
CPGBNV	1	248	5.90E-30	Tospo_nucleocap	IPR002517
	261	271	NA	LC	
NSGBNV	1	437	2.30E-194	Bunya_NS-S_2	IPR004915
NPSGBNV	39	283	2.70E-26	Pfam:3A	IPR000603
EGPGBNV		1,014	1.00E-23	Bunya_G1	IPR005167
	4	26		TMR	
	294	313	NA		
	318	340	NA		
	407	429	NA		
	1,048	1,070	NA		
	44	62	NA	LC	
	133	151	NA		
RDRPGBNV	834	1673	4.10E-199	Bunya_RDRP	IPR007322
	395	407	NA	LC	
	608	621	NA		
	2,292	2,302	NA		
	2,690	2,701	NA		
	2,849	2,858	NA		
MPGBNV	23	264	1.60E-25	3A	IPR000603

(B)							
Domain	From	To	e-Value	Domain	From	To	e-Value
Pfam:PCNA_N	1	125	4.00E-58	PDB:2ZVW|H	1.00E+00	2.55E+02	1.00E-165
Pfam:Rad9	12	248	6.90E-08	SCOP:d1axca2	1.27E+02	2.55E+02	5.00E-67
Pfam:PCNA_C	127	254	1.20E-61				
CDK1							
CKS	3.00E+00	7.20E+01	4.90E-46	SCOP:d1puc__	1.00E+00	7.00E+01	3.00E-35
Low complexity	7.40E+01	8.30E+01	N/A	Blast:CKS	3.00E+00	7.20E+01	2.00E-46
				PDB:2AST|C	3.00E+00	7.10E+01	3.00E-32
CAM							
EFh	1.20E+01	4.00E+01	1.24E-06	PDB:2L1W|A	2.00E+00	1.50E+02	1.00E-104
EFh	4.80E+01	7.60E+01	3.37E-08	SCOP:d1exra_	5.00E+00	1.48E+02	2.00E-41
EFh	8.50E+01	1.13E+02	6.29E-08	Blast:FBG	8.00E+00	1.48E+02	2.00E-23
EFh	1.21E+02	1.49E+02	2.56E-08				
GSK1							
S_TKc	7.40E+01	3.58E+02	7.03E-92	PDB:3L1S|B	2.70E+01	4.05E+02	1.00E-175
				SCOP:d1h8fa_	5.70E+01	4.04E+02	1.00E-150
				Blast:S_TKc	7.40E+01	3.58E+02	0.00E+00
HSP70							
Pfam:HSP70	8.00E+00	6.17E+02	########	PDB:3C7N|B	5.00E+00	5.59E+02	0.00E+00
Pfam:MreB_Mbl	1.18E+02	3.86E+02	9.30E-16	Blast:DYNc	1.02E+02	1.26E+02	3.00E-04
Low complexity	6.27E+02	6.39E+02	N/A	SCOP:d1bupa2	1.96E+02	3.86E+02	2.00E-88
				SCOP:d1dkza_	3.96E+02	6.15E+02	1.00E-102
Cul1							
CULLIN	4.14E+02	5.63E+02	6.29E-75	SCOP:d1ldja2	9.00E+00	3.79E+02	1.00E-124
Cullin_Nedd8	6.65E+02	7.32E+02	4.55E-32	PDB:4A0K|A	2.40E+01	7.38E+02	1.00E-132
				SCOP:d1ldja3	3.81E+02	6.50E+02	7.00E-96
				Blast:CULLIN	4.14E+02	5.63E+02	1.00E-103
				SCOP:d1ldja1	6.57E+02	7.38E+02	1.00E-35
TCP							
Low complexity	3.70E+01	5.30E+01	N/A	SCOP:d1jdqa_	1.36E+02	1.50E+02	2.90E-01
Low complexity	6.20E+01	7.00E+01	N/A				
Low complexity	1.34E+02	1.43E+02	N/A				
LOX1							
LH2	1.10E+01	1.74E+02	3.92E-71	PDB:2IUK|B	5.00E+00	8.59E+02	0.00E+00
Pfam:Lipoxygenase	1.85E+02	8.42E+02	0.00E+00	Blast:LH2	1.10E+01	1.74E+02	1.00E-110
				SCOP:d1f8na1	1.78E+02	8.59E+02	0.00E+00

**TABLE 2:** Physicochemical properties of the A) GBNV proteins and B) soybean proteins.

(A)												
Given name	Accession	Description	Protein Len (aa)	PI	MW (Da)	II	U/S	AI	GRAVY	Serine (S)	Threonine (T)	Tyrosine (Y)
CPGBNV	XDU23736	Coat protein partial [groundnut bud necrosis virus]	271	8.79	29972.76	42.45	U	78.52	−0.237	22	8	6
NSGBNV	AWV56699	NSs [groundnut bud necrosis virus]	439	6.89	49606.66	33.63	S	90.77	−0.224	31	11	6
NSPGBNV	AWV56697	Nonstructural protein [groundnut bud necrosis virus]	307	8.46	34228.04	45.05	U	85.73	−0.392	21	8	3
EGPGBNV	AWV56696	Envelope glycoprotein [groundnut bud necrosis virus]	1,121	5.95	127242.2	37.92	S	88.98	−0.172	70	27	18
RDRPGBNV	AWV56695	RNA-dependent RNA polymerase [groundnut bud necrosis virus]	2.878	7.78	331318	40.35	U	88.43	−0.3	176	74	41
MPGBNV	UVD61612	Movement protein, partial [groundnut bud necrosis virus]	286	7.8	32000.53	45.85	U	87.59	0.371	21	10	4

(B)																				
Description	Protein Len (aa)	PI	MW (Da)	II	U/S	AI	GRAVY	Location	TMH	N-T/SP/mTP/ cTP/TTP	iPSORT	Ch	Exon	Cul	From	To	Total len	Serine (S)	Threonine (T)	Tyrosine (Y)
Proliferating cell nuclear antigen [*Glycine max*]	266	4.68	29,521.72	42.22	U	88.65	−0.131	nucl: 11, cyto: 2, chlo: 1	N	O	No	8	4	Williams 82	44,583,455	44,585,532	4,72,27,184	19	10	3
Cyclin-dependent kinases regulatory subunit 1 [*Glycine max*]	103	9.34	12,363.13	62.16	U	87.96	−0.706	cyto: 5.5, cyto_E.R.: 3.5, nucl: 3, extr: 3, chlo: 1, cysk: 1	N	O	No	12	-	Hwangkeum	-	-		5	0	3
Calmodulin [*Glycine max*]	150	4.04	17,017.94	36.38	S	81.87	−0.47	chlo: 5, cyto: 5, extr: 2, cysk_nucl: 1.33333, cysk_plas: 1.33333	N	O	No	10	4	Williams 82	41,275,605	41,277,474	5,16,38,687	6	7	1
Glycogen synthase kinase-3 [*Glycine max*]	410	8.67	46,431.82	35.2	S	91.71	−0.231	cyto: 10, nucl: 1, extr: 1, cysk: 1, E.R._vacu: 1	N	O	No	10	13	Williams 82	38,104,554	38,108,942	5,16,38,687	9	9	6
Heat shock 70 kDa protein [*Glycine max*]	645	5.27	70,865.58	33.92	S	83.02	−0.411	cyto: 11, cysk: 2, chlo: 1	N	O	No	17	2	Williams 82	5,663,501	5,666,085	4,17,40,656	19	19	7
Cullin-1 [*Glycine max*]	738	6.63	86,171.97	45.21	U	87.85	−0.455	cyto: 9, nucl: 3, plas: 1, golg: 1	N	O	No	19	21	Williams 82	46,881,575	46,887,169	5,12,72,880	21	26	14
TCP family transcription factor, partial [*Glycine max*]	155	10.17	17,760.05	67.23	U	61.03	−1.205	nucl: 11, extr: 2, chlo: 1	N	O	No	-	-	Hwangkeum	-	-		11	6	0
Lipoxygenase [*Glycine max*]	839	6.29	96,300.6	34.9	S	88.65	−0.306	cyto: 11, chlo: 2, pero: 1	N	O	Mit	-	-	-	-	-		43	35	10

AA, amino acids; Cyt, cytoplasm; Chl, chloroplast; Mit, mitochondrion; PI, isoelectric point; MW, molecular weight; II, instability index; AI, aliphatic index.

**FIGURE 2:**
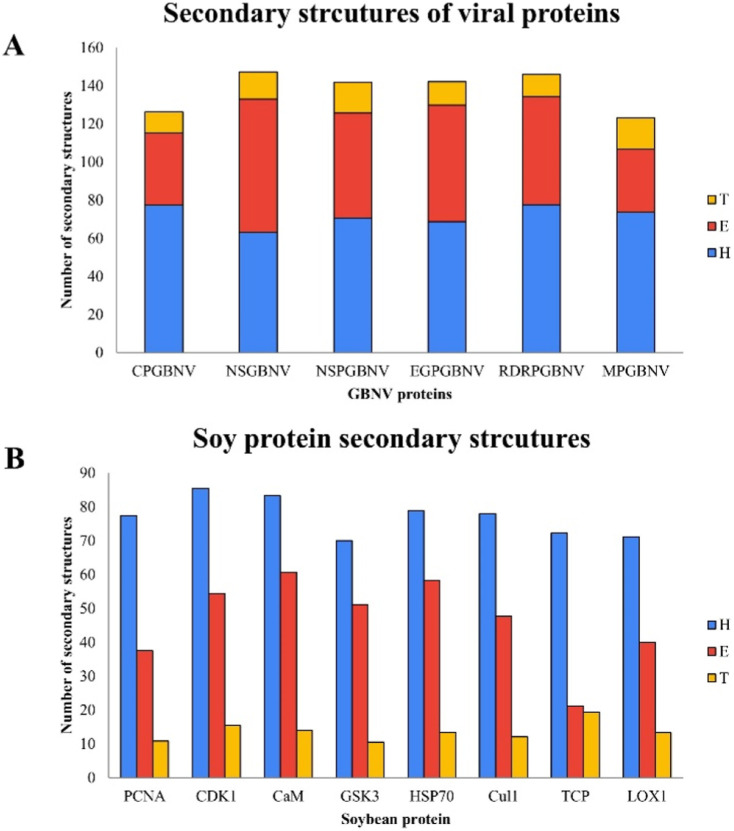
Pictorial representation of secondary structures, namely, helices (red), turns (blue), and coils (yellow); **(A)** GBNV proteins and **(B)** soybean proteins (H - alpha helix, T - turns and E - Beta sheets).

The secondary structures of the proteins were also studied. All the proteins contain alpha helices, beta-sheets, and turns (Figure 2). Based on Figures 2A,B, it can be observed that both the protein groups have comparable helices and turns, while there is a variation in the β-sheets, ranging from 32.9 to 69.9 in the virus and 21.3 to 60.7 in the soybean proteins. For the β-sheets, CDK1 (85.4) and CP (77.5) have the highest percentages.

### Hydrophobicity plot

**FIGURE 3:**
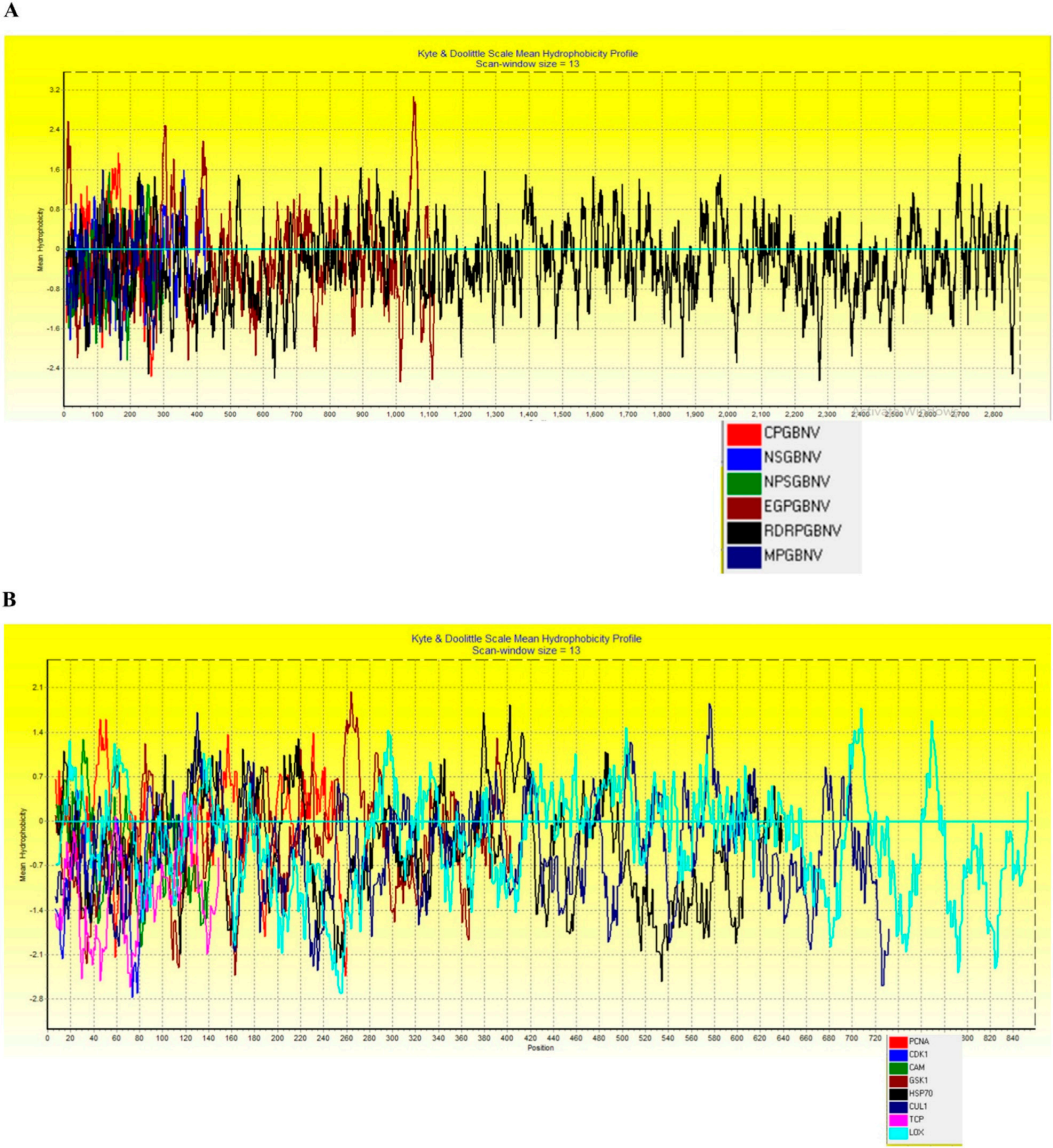
Hydrophobicity plot of the **(A)** viral and **(B)** host proteins.

The proteins showed that all of them contain hydrophobic and hydrophilic regions (Figure 3). It was observed that all of the sequences are more hydrophilic (peaks toward the negative end) in nature as compared to the hydrophobic (peaks toward the positive end) regions. Different regions along the protein sequence show a high degree of hydrophobicity (positive scores). There are also regions that show a high degree of hydrophilicity (negative scores). Since all proteins span the cytoplasm along with other cell organelles, the plot has both hydrophobic and hydrophilic regions. Since the hydropathicity profile shows an alternating pattern of hydrophobic and hydrophilic regions, this indicates a potential modular structure of the protein, with different domains or regions that have different functional properties.

### Motifs and conserved domain analyses

Conserved domains were analyzed using MEME. A total of 10 conserved domains were identified from both the viral and host protein sequences. From this, it could be understood that a large number of motifs are conserved in all the proteins. The putative functions of the motifs were analyzed using the NCBI-CDD. For the viral proteins, it was found that two of the 10 motifs belong to the 3A/RNA2 movement protein and the bunyavirus glycoprotein G1 superfamily, while the other eight motifs could be novel as no function was identified (Table 3). Similarly, for the soy proteins, the results of the CDD analysis showed that the sequences contained the cyclin-dependent kinase and the cullin superfamily, and the remaining motifs had no annotated function assigned (Table 3). Hence, all the motifs need to be studied to further understand their roles in abiotic and biotic stresses.

**TABLE 3:** Conserved motifs of A) GBNV proteins and B) soybean proteins are shown in the logo form.

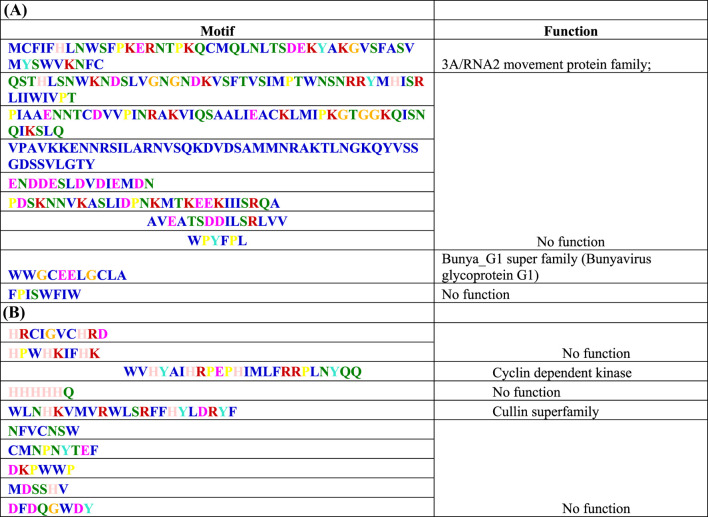

### Chromosome localization

**FIGURE 4:**
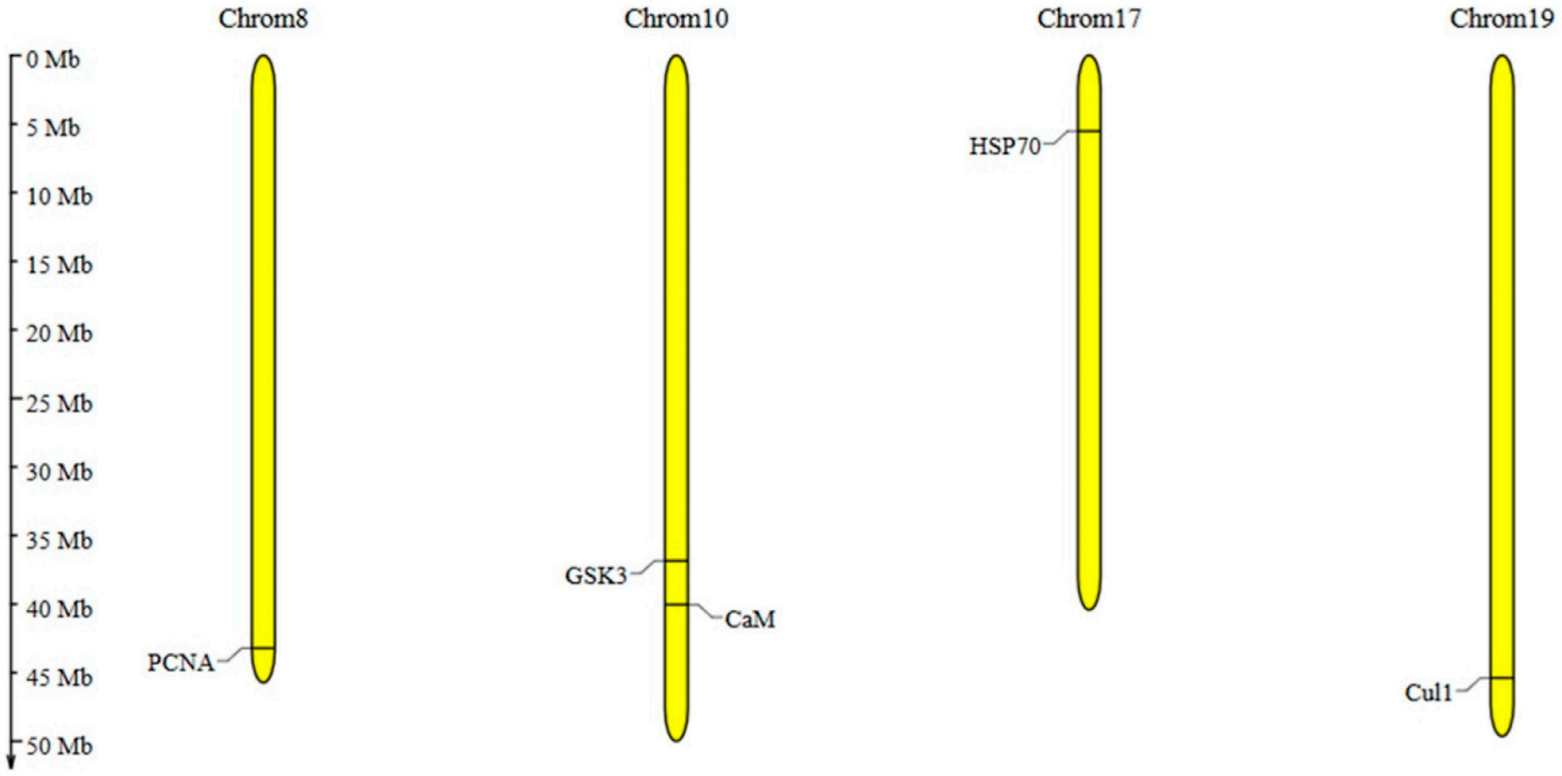
Chromosomal distribution of host genes.

A graphical representation of the genes whose information was available was plotted on the chromosomes to understand their respective positions (Figure 4).

### Gene structure analysis: exon–intron structure

**FIGURE 5:**
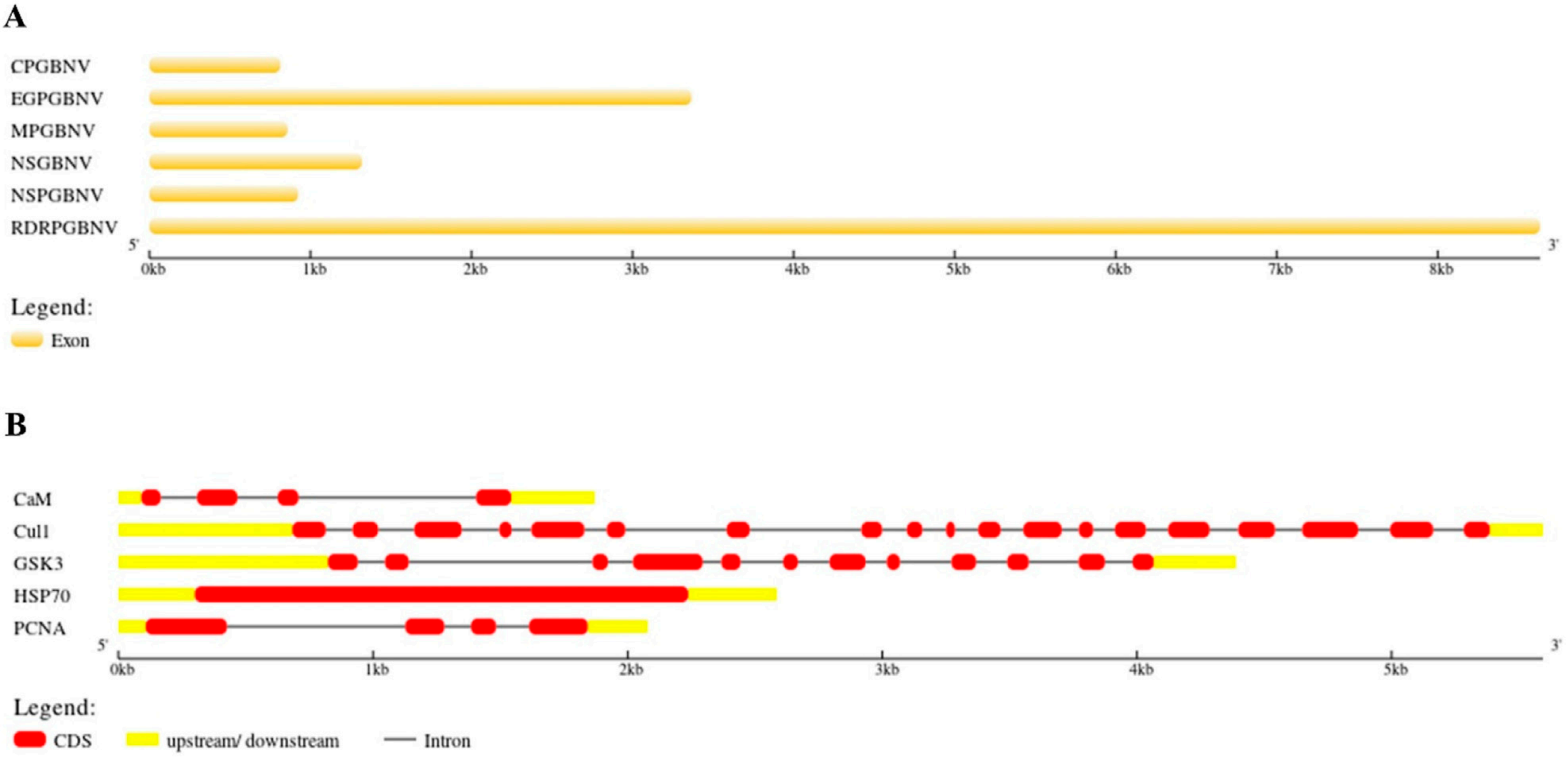
Exon–intron structure of the **(A)** GBNV proteins and **(B)** soybean proteins. Exons are represented in orange and red, respectively; yellow boxes show untranslated regions, and the black line shows intronic regions.

The gene structure analysis was carried out using the sequences whose coding and gene sequences were available. It is known that RNA viruses do not contain intronic regions (Šimičić and Židovec-Lepej, 2022); therefore, Figure 5A does not show any intronic regions. On the contrary, eukaryotic genomes contain both intronic and exonic regions, which undergo processing during transcription, wherein the introns are removed for translation (Figure 5B). It is interesting to note that the genes contain at least two intronic regions. It is also notable that all the sequences contain the long 5ʹ untranslated regions (UTRs). CaM and PCNA contain four exons and three introns; Cul1 contains the highest number of exons (19) and introns (18), followed by GSK3, with 12 exons and 11 introns, while HSP70 has no introns and only a single long exonic region.

### Calmodulin motif prediction

**FIGURE 6:**
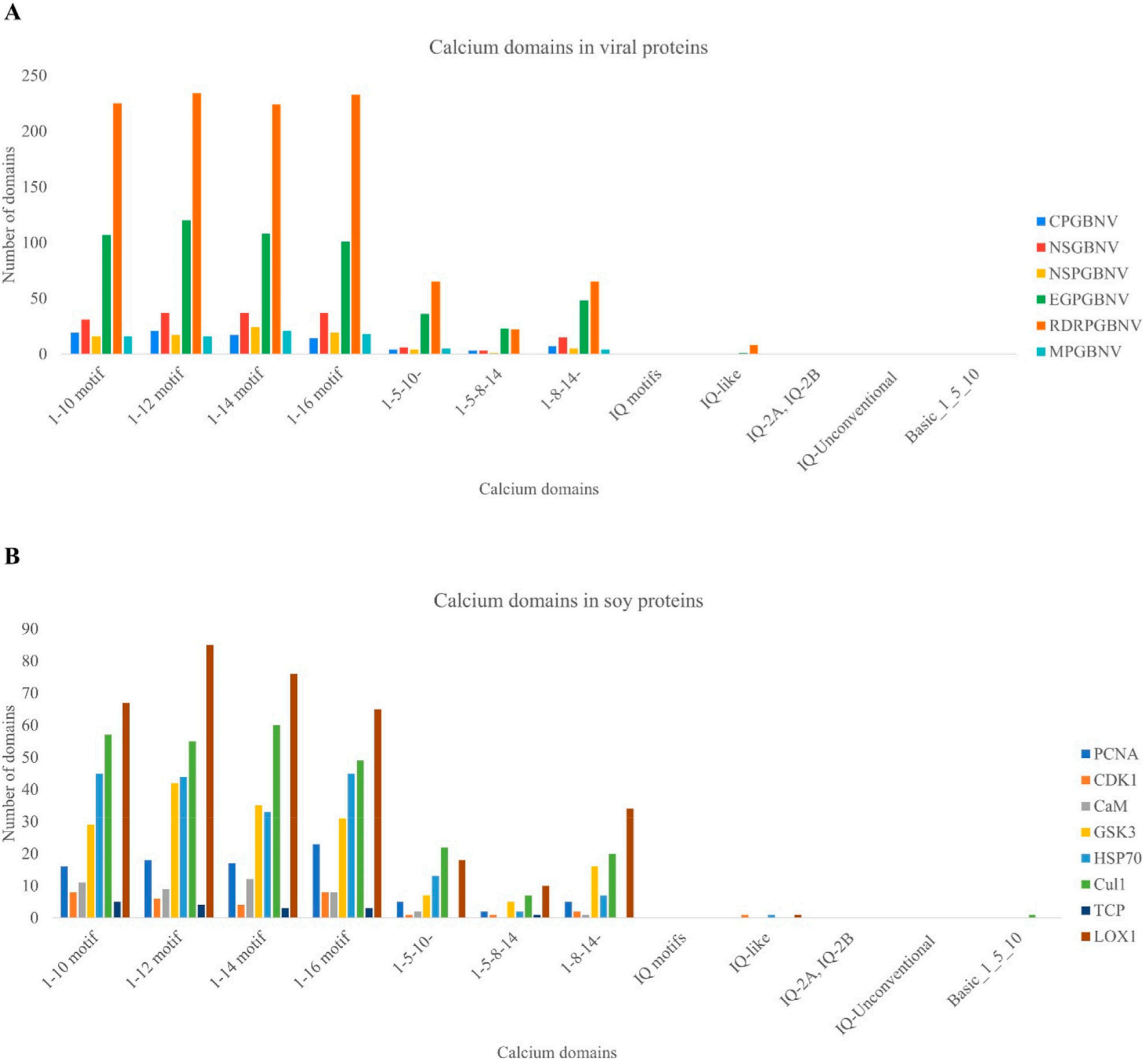
Putative calmodulin-binding motifs are present in the **(A)** viral and **(B)** host sequences.

Calmodulin databases used to identify the motifs were IQ, IQ-like, IQ-2A, IQ-2B, IQ-unconventional, 1–10 motif, 1–12 motif, 1–14 motif, and 1–16 motif. Here, none of the viral proteins contain IQ, IQ-2A, IQ-2B, IQ-Unconventional, and Basic_1_5_10; however, the EG and RDRP have one and eight, respectively, of IQ-like motifs. Similarly, in the host proteins, IQ, IQ-2A, IQ-2B, IQ-Unconventional, and Basic_1_5_10 motifs were absent. However, CDK1, HSP70, and LOX1 contain one IQ-like motif, and Cul1 contains one Basic_1_5_10 motif. The other motifs (1–10 motif, 1–12 motif, 1–14 motif, and 1–16 motif) are present in abundance in all the viral and host proteins (Figure 6). Similarly, calmodulin-binding site prediction showed that EG contains two sites, while the remaining viral and host proteins have only one binding site. On the other hand, CaM has no binding site as it is itself a calmodulin protein (Figure 6; Table 4).

**TABLE 4:** Putative calmodulin binding sites present in the A) viral and B) host sequence sites predicted using the calmodulin target database scores, where those having a score of more than 9 are shown in bold.

(A)	
Proteins	Sites
CPGBNV	178 K**KEKLGIKNF**ST 188
NSGBNV	4 **ARSAASEFVKSYGTRDNRAI** 23
NSPGBNV	206 E**KYAKGVSFASVMYSWVK**N 224
EGPGBNV	541 G**QSFKGNLTSLKIAN**R 555 1066 I**FKLSRTYYVDK**R 1079
RDRPGBNV	272 A**RQMRLTKK**K 279
MPGBNV	188 E**KYAKGVSFASVMYSWVK**N 204

(B)	
Proteins	Sites
PCNA	1 **MLELRLVQGSLLKKVLESVK** 20
CDK1	42 W**RAIGVQQSRGWV**HYAIHRP 62
CaM	none
GSK3	**101 AIKKVLQDKRYKNRELQT**MR 121
HSP70	261 N**ARALRRLRTACERAKRTLSST** 282
Cul1	531 CLEVFK**GFYETRTKHRKL**TW 551
TCP	**20 LVAARERMKLK RTQKEPAKI 39**
LOX1	158 **VYNFKSYKKNRIFF** 172

### Multiple sequence alignment and phylogenetic analysis

**FIGURE 7:**
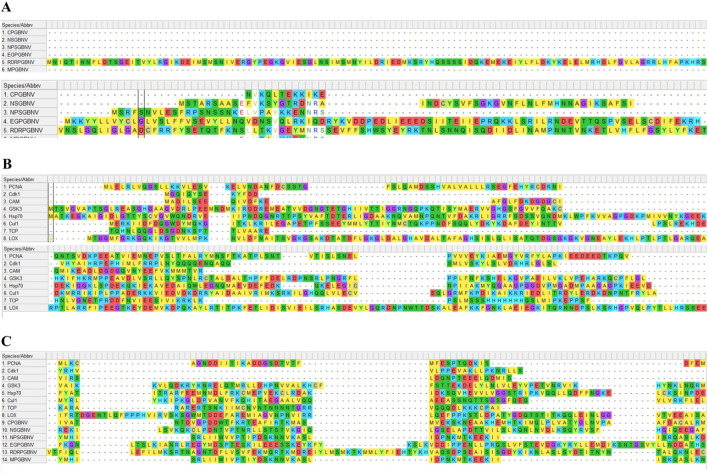
A portion of multiple sequence alignment of **(A)** viral, **(B)** host, and **(C)** combined sequences.

**FIGURE 8:**
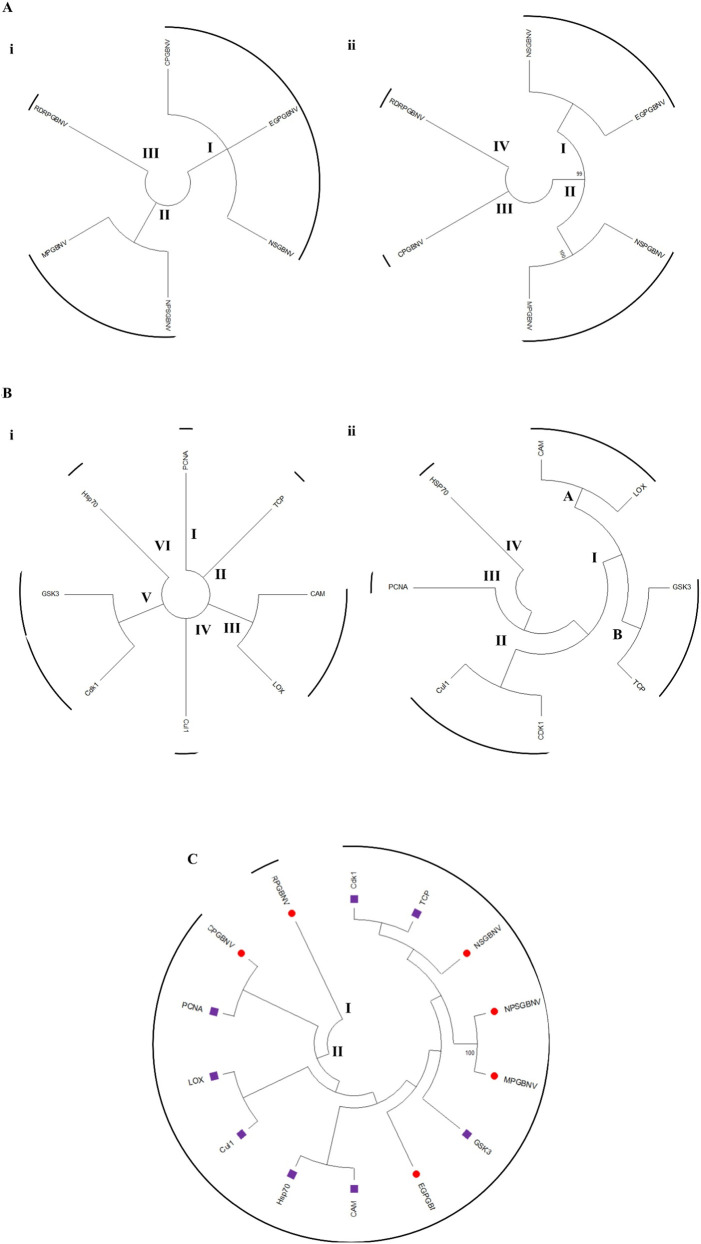
Phylogenetic tree of the **(A)** viral proteins’ i) protein and ii) gene, **(B)** host proteins’ i) protein and ii) CDS, and **(C)** all proteins. The tree was constructed by using the NJ method using MEGA12. The number of bootstrap values was 1,000 replicates (only values >80 are indicated) with the root RDRP and HSP70.

From the alignment, it is very clear that there are no as such conserved regions, indicating the divergent nature of the proteins in both groups. From the alignment, it is evident that the RDRP of the six viral proteins and LOX1 from the host proteins have longer sequences, thereby leading to an asymmetric alignment at most of the places in the alignment, especially having an initial stretch of unaligned amino acids (Figures 7A–C). The phylogenetic analyses of the viral genes and proteins and host CDS and proteins revealed interesting observations. For the viral genes, there are four clades (I–IV), and the proteins’ phylogeny consisted of three clades only, from I to III. On the contrary, the host protein sequences’ phylogeny revealed the presence of six clades (I–VI), and the CDS tree showed four clades (I–IV), with clade I having two subclades (A and B). The combined protein phylogeny revealed only two major clades, with clade II containing subclades. HSP70 and RDRP were considered the root of the trees (Figures 8A–C). WebLogo was also constructed for the viral and soybean proteins (Supplementary Figure S2).

### Structure prediction: homology modeling

**FIGURE 9:**
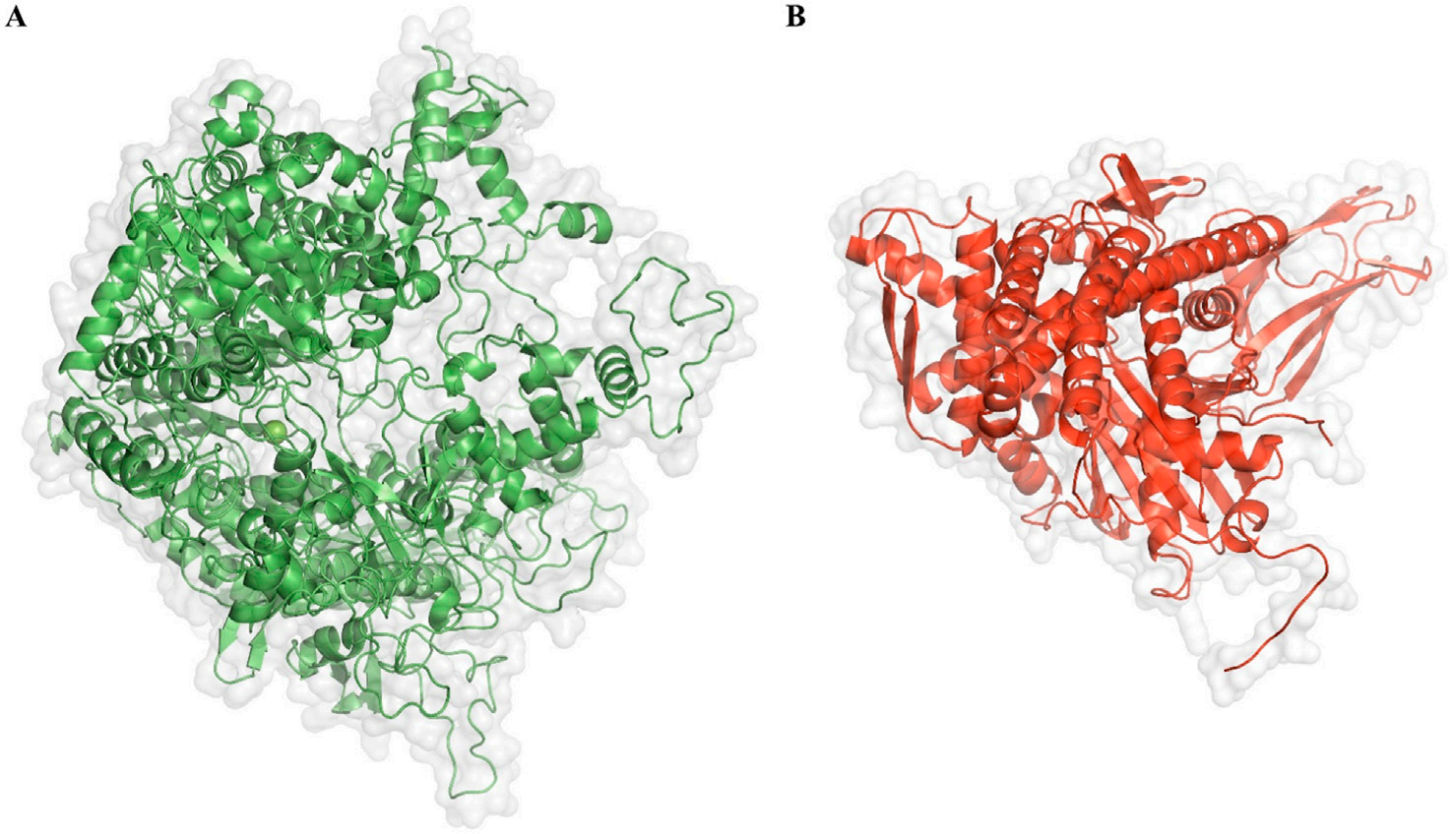
**(A)** RDRP and **(B)** HSP70 protein structures predicted using the SWISS MODEL.

As the template was not available for any of the proteins, their structures were predicted. For the prediction of models of the viral and host proteins, the FASTA sequences were subjected to both SWISS-MODEL and Phyre2.2. Both these tools work on the principle of homology modeling. HM is the most accurate computational method used to create reliable structural models and is commonly used in many biological applications. HM is used to predict the 3-dimensional (3D) structure of a protein via sequence alignment with the template proteins through the repository (Schwede et al., 2003). The structures of all the proteins were predicted along with their 3D models using the existing templates in the database and measuring the Qmean score (Guex et al., 2009; Bienert et al., 2017; Studer et al., 2020) for further analyses. SWISS-MODEL uses a query sequence to generate the best match with the template through net-forming BLAST (Altschul et al., 1997) and HHblits (Remmert et al., 2012). The model was selected based on the maximum global model quality estimate (GMQE) and percent identity (closest alignment) (Biasini et al., 2014) based on the models that were built (Table 5). The template 8ki7 was considered the best match for the RDRP based on its similarity percentage (51.64), rather than model 1 (8ki9), which had a similarity percentage of only 47.64. Likewise, the template A0A6P5U5G1 was considered the best match for the HSP70 (Figure 9), while Phyre2.2 yields a model based on the coverage and maximum confidence scores (Table 5).

**TABLE 5:** Model validation scores of the A) viral and B) host proteins.

Viral proteins	SWISS-MODEL			PROCHECK				ProSA	SAVES		Phyre2.2	
	Template (PDB ID)	Identity (%)	GMQE	Most favored regions (%)	Additional favored regions (%)	Generously favored regions (%)	Disallowed regions (%)	ProSA Z-score	ERRAT overall quality (%)	Verify 3D averaged 3D–1D score >0.1 (%)	Confidence	Coverage
CPGB	5ip1	30.2	0.68	91.2	7.5	1.3	0	−6.75	96.72	57.33 (F)	100	90
EGPGB	2a57	23.23	0.24	86.4	12.6	0.9	0	−5.88	68.98	53.52 (F)	100	34
MPGB	2iml	20.31	0.07	92.9	7.1	0	0	−2.72	100	7.03 (F)	59.2	15
NPSGB	1hwg	18.52	0.08	87.3	10.1	1.3	1.3	−1.22	72	15.91 (F)	55.6	14
NSGB	4r7u	13.04	0.07	81	16.7	2.4	0	−1.08	87.5	21.74 (F)	25.8	21
RDRPGB	8ki7*	51.64	0.5	81	15.6	2.2	1.2	−10.35	84.84	41.16 (F)	100	69

Host proteins	SWISS-MODEL			PROCHECK	ProSA	SAVES		Phyre2.2	
	Template (Uniprotkb ID)	Identity (%)	GMQE	Most favored regions (%)	ProSA Z-score	ERRAT overall quality (%)	Verify 3D averaged 3D–1D score >0.2 (%)	Confidence	Coverage
CAM	Q39890	100	0.83	93.5	−7.01	95.04	71.33 (F)	99.8	99
CDK1	A0A6P3Z777	94.38	0.79	89.7	−5.37	92.6	78.65 (F)	100	69
Cul1	1NB34	100	0.93	94.8	−14.07	97.6	83.88 (P)	100	95
GSK3	P51137	94.39	0.87	91.2	−9.72	94.2	70.98 (F)	100	86
HSP70	A0A6P5U5G1	91.43	0.89	92.9	−12.6	95	97.53 (F)	100	85
LOX	A0A0R0IYE6	99.77	0.92	91.1	−12.62	95.56	86.38 (P)	100	96
PCNA	1N036	98.87	0.94	94.2	−8.16	100	75.94 (F)	100	96
TCP	S4WUR0	100	0.57	60	−0.08	50	0.00 (F)	3.5	15

* Model 2 is selected instead of model 1 (8ki9) based on the sequence identity of 47.46%.

### Model validation: Ramachandran plot and structure analysis

**FIGURE 10:**
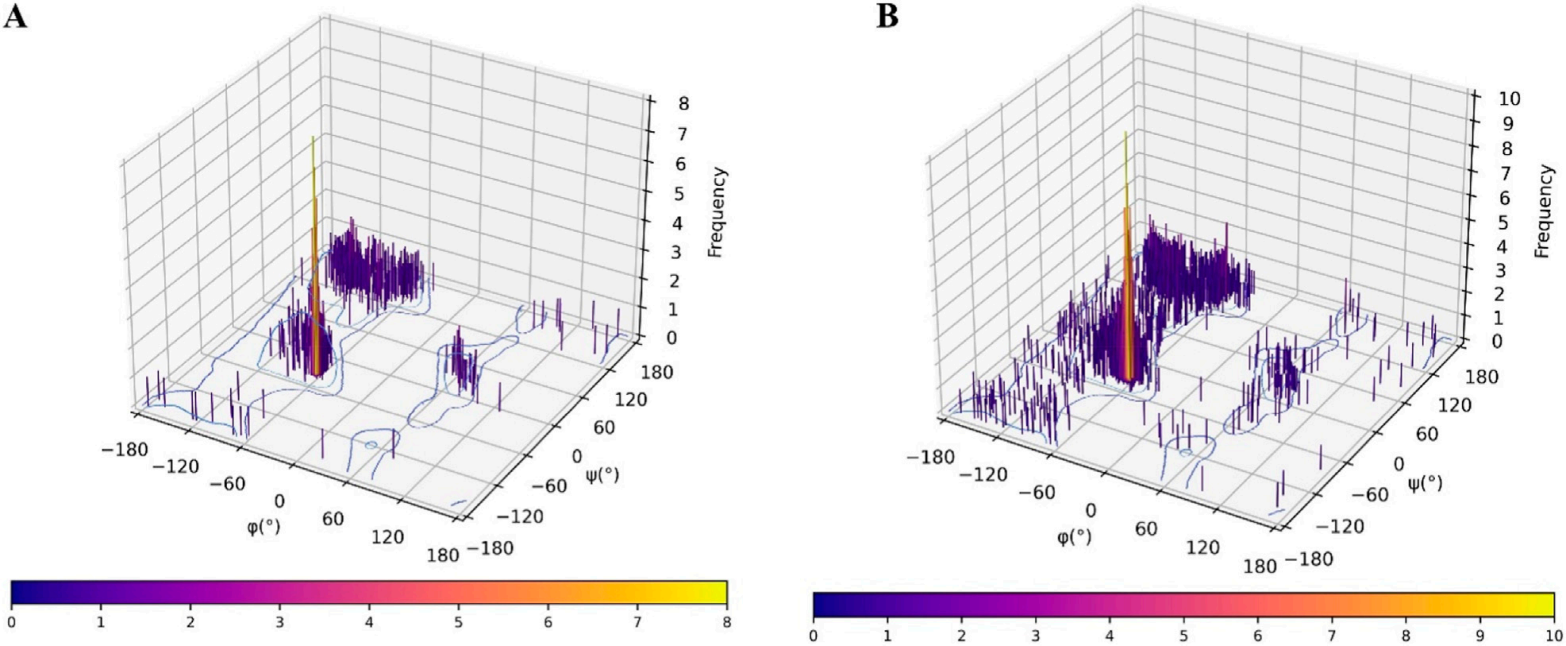
3D Ramachandran plots of **(A)** HSP70 and **(B)** RDRP. The bars represent the frequency of torsion angles.

**FIGURE 11:**
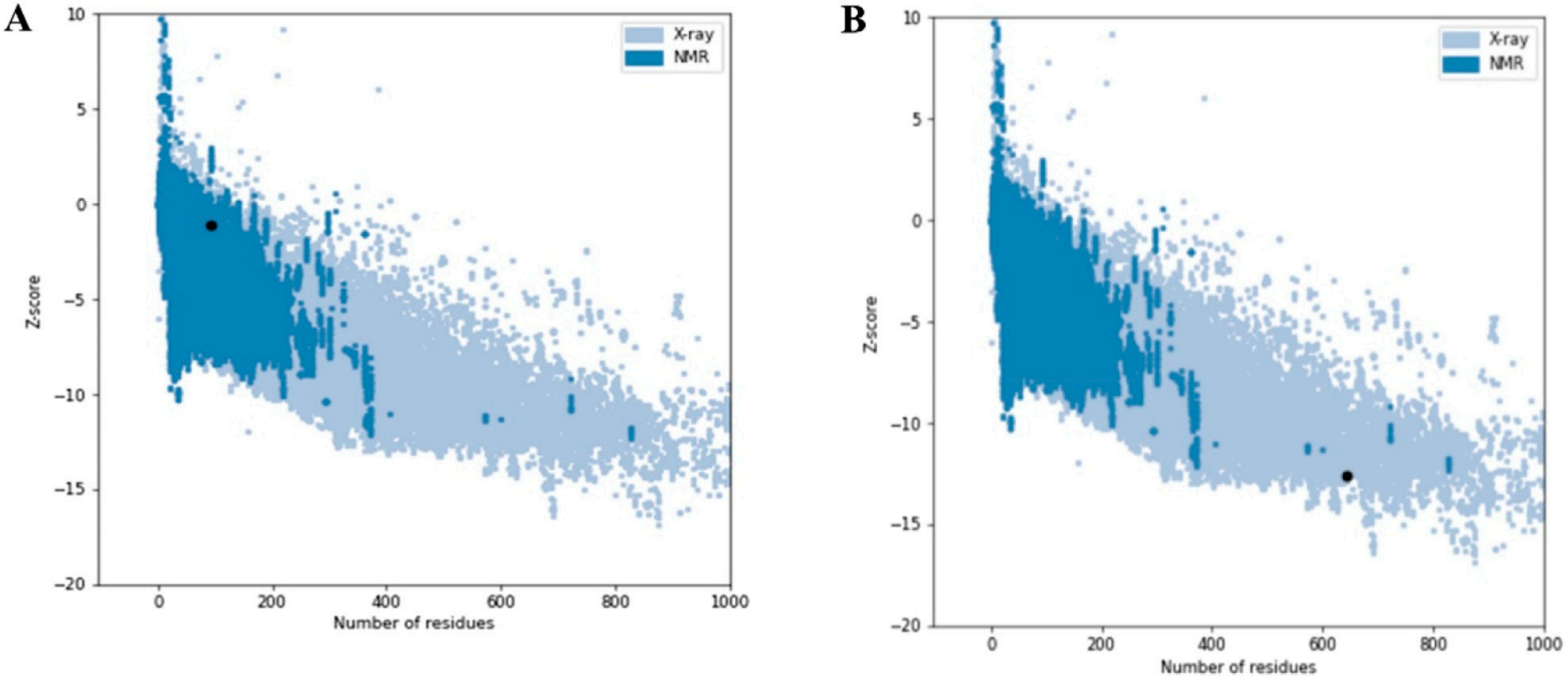
Graphical representation of z-scores of **(A)** RDRP and **(B)** HSP70. The black dot represents the z-score of the protein of interest in a plot containing all the z-scores that have been experimentally determined and are currently available in the PDB. The X-ray and NMR structures are shown in light and dark blue, respectively.

The selected best models were validated using PROCHECK of SAVES and the PDBSum Generator to assess the stereochemical consistency and residual geometry of the 3D model, as well as its overall geometry through RC plots (Table 5). It also generated RC plots of the models (Supplementary Figure S3). It was also used to determine whether the amino acid sequence of the atomic model is compatible. RC plot analysis using PROCHECK showed that more than 80% of the residues are in the favorable regions for all the viral proteins, whereas for the host proteins, more than 90% fall in the favorable regions, except for the TCP (60%) (Table 5). To further confirm, RC plots were constructed through PyMol and Rama plot as well for structural integrity assessment of RDRP and HSP70 (these proteins are selected as the best PPI through docking; see the section *Docking* for further details) (Ho and Brasseur, 2005; Kumar and Rathore, 2025). Most of the amino acid residues of HSP70 and RDRP are clustered in the core regions of the RC plot, thus indicating favorable phi and psi angles and stability of the protein. For the RDRP, the disallowed percentage of residues was found to be 2.789%, and in the case of HSP70, it was 1.244% (Figure 10). The energy potential of the predicted models was computed using ProSA through their z-scores. The highest z-score was −10.35 for RDRP (Figure 11) and −14.07 for Cul1. The more negative the value is, the better the model is. Therefore, all the predicted models are of good quality (Table 5; Supplementary Figure S4). The ERRAT validation showed that for the viral proteins, four proteins (CP, MP, NS, and RP) scored above 80, while EG and NP scored 68.98% and 72%, respectively. Similarly, for the host proteins, all the proteins scored above 90%, except for the TCP (50%) (Table 5). As shown by Verify3D, the movement protein of the virus and soybean TCP were found to be least compatible in their 3D model with their 1D aa sequences, as compared to other proteins.

### Energy minimization and active site identification of the proteins for docking

EM is a crucial step in the process of docking for having lower energy states, optimizing geometries, and removing steric hindrances (Mirzaei et al., 2012). Therefore, it is essential to minimize the energies for better docking. The EM of the proteins and compounds was subjected to the YASARA tool (Supplementary Table S1). Proteins contain active sites or pockets wherein the ligands (synthetic or natural compounds) and other proteins bind, thereby initiating either activation or repression (regulation) of the proteins (Singh et al., 2011). In this study, the active sites were identified using the SPIDDER and confirmed by the AADS server. The key sites of the proteins are provided in Supplementary Table S2.

### Docking: protein–protein and protein–ligand interactions

**FIGURE 12:**
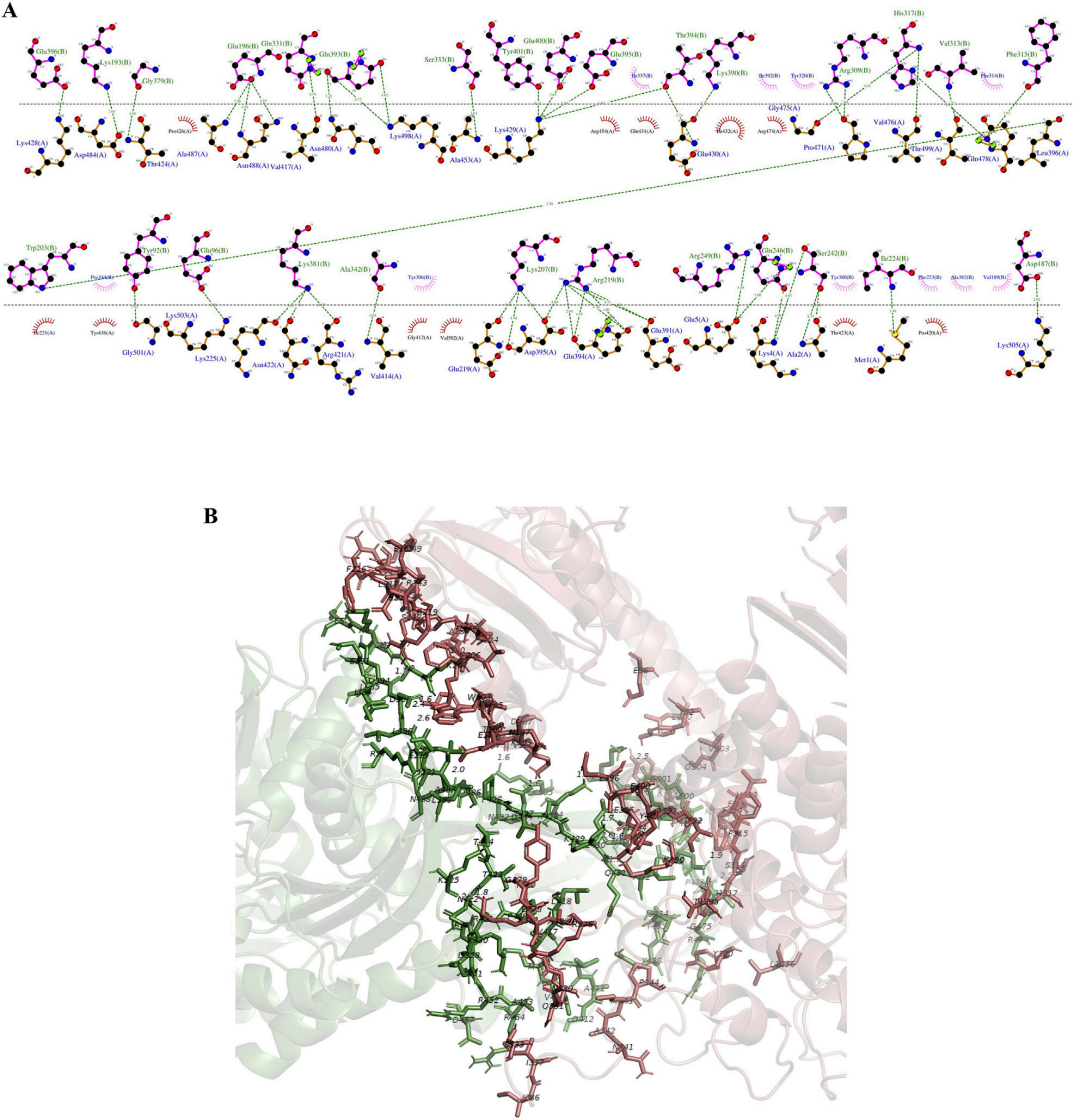
**(A)** LigPlot representations of the interactions of RDRP with residues of HSP70 (H-bond/electrostatic interactions are shown in green, and the residues which are involved only in hydrophobic interactions are represented by red semicircles). **(B)** Zoomed-in view of the sites of the RDRP and HSP70 (protein ribbons are shown in green and red, respectively, and interacting residues are shown in sticks).

For each of the viral proteins, all viral proteins were docked with the host proteins, and their HADDOCK and z scores and RMSD values were interpreted. Therefore, among the 50 (six virus × eight host +two RDRP-HSP70 dockings) PPIs (Supplementary Table S3), those having the highest negative scores of HADDOCK and other parameters were selected for visualizing the poses. Based on the scores, binding energies, and statistics, the best PPIs from each of the combinations were chosen (Table 6). Apart from these, electrostatic energies and van der Waals forces also play crucial roles in PPIs. Electrostatic energies and van der Waals forces of attraction help in providing the stability and protein–protein binding in cases of hetero-complexes (Roth et al., 1996; Talley et al., 2008). Based on these parameters, the best PPI was determined to be RDRP-HSP70_I; however, the HADDOCK score was positive (90.1 ± 19.5), which could be because not all active sites were involved in the interactions; therefore, the HADDOCK refinement was formed, and other active sites of RDRP were also docked (RDRP-HSP70_II and RDRP-HSP70_III). The refined docking resulted in both restraint violation energy and a z-score of 0.0. The interactions are shown in Figure 12.

**TABLE 6:** Statistical scores of the best protein–protein interactions from all the dockings performed through HADDOCK.

PPI	Cluster size	Binding energy (kcal/mol)	HADDOCK score	RMSD	Van der Waals energy	Electrostatic energy	Desolvation energy	Restraints violation energy	Z-score	Inhibition constant (M)
CP_PCNA	23	−9.6	−40.2 ± 3.7	22.9 ± 3.7	−60.3 ± 4.5	−581.2 ± 32.8	23.8 ± 3.9	1125.1 ± 35.5	−1.8	9.02271 $\times 10^{-8}$
EG_Cul1	6	−15.7	−2.7	1.2 ± 0.7	−104.8 ± 24.8	−455.6 ± 57	−11.2 ± 4.7	2044.4 ± 178.3	−2.4	3.01329 $\times 10^{-12}$
MP_CDK1	16	−16.1	−104.4 ± 10	1.1 ± 0.6	−82.2 ± 9	−371.6 ± 14.7	3.8 ± 2.8	483.5 ± 69.3	−2.7	1.5329 $\times 10^{-12}$
NPS_CDK1	13	−16.9	−120.2 ± 24.4	1.2 ± 0.9	−114.7 ± 8.9	−328.9 ± 44.9	−24.4 ± 3.2	846.1 ± 119.7	−2	3.96696 $\times 10^{-13}$
NS_CAM	20	−13.8	−58.9 ± 9.2	20.9 ± 0.2	−92 ± 2.5	−427.1 ± 15.4	−8.8 ± 3.5	1273.8 ± 100.3	−2.7	7.46959 $\times 10^{-11}$
RD_HSP70	55	−19.5	90.1 ± 19.5	1 ± 0.6	−122.7 ± 4.9	−547.3 ± 131	−18.4 ± 7.5	3407.181.3	−2.4	4.90375 $\times 10^{-15}$
RD_HSP70_refined	20	−12.12	−302.3 ± 3.1	0.7 ± 0.4	−146.9 ± 2.7	−673.4 ± 21.6	−20 ± 4	0 ± 0	0	1.28E-09

**FIGURE 13:**
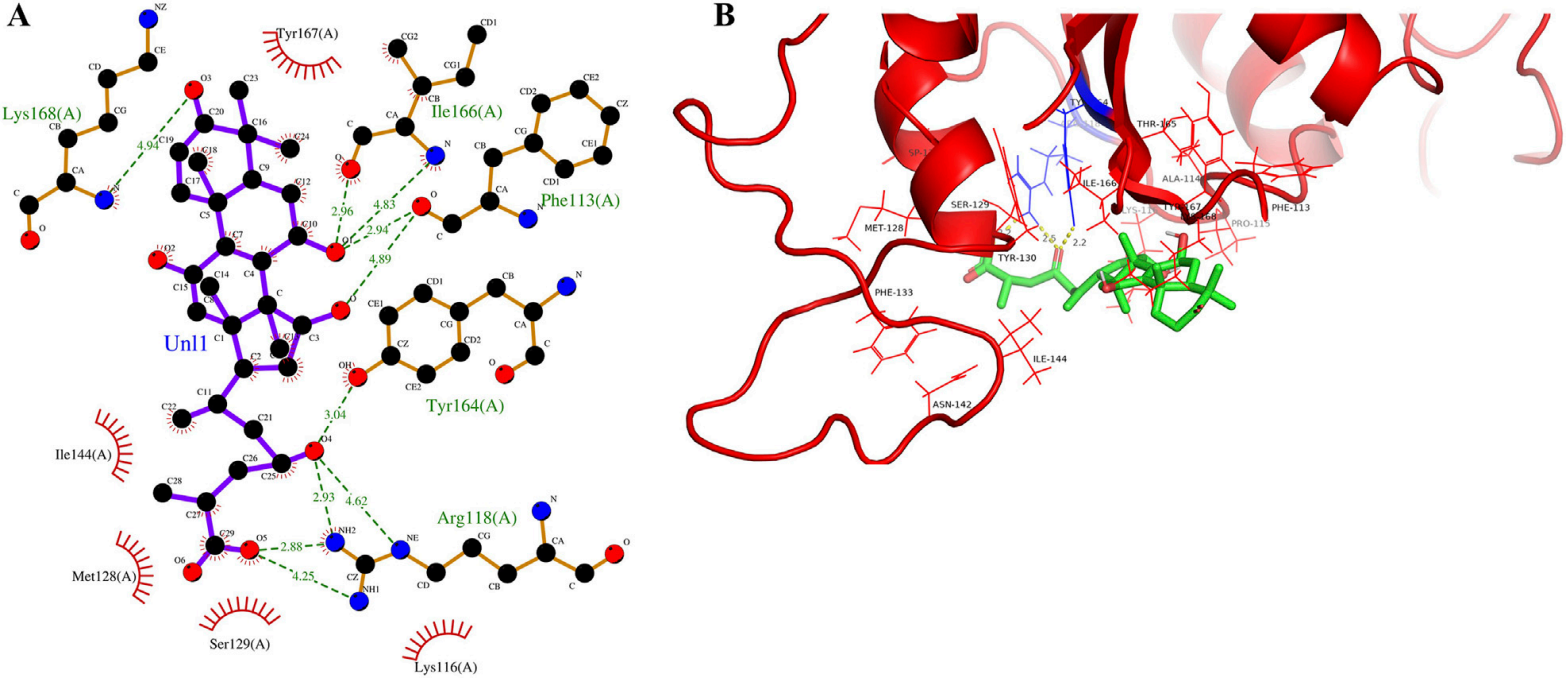
**(A)** LigPlot representations of the interactions of ganoderic acid A (GAA) with residues of RDRP (H-bond/electrostatic interactions are shown in green, and the residues that are involved only in hydrophobic interactions are represented by red semicircles). **(B)** Zoomed-in view of the interacting sites of the GAA and RDRP (protein ribbons are shown in red, polar interactions are shown as sticks with residues in blue, and the bound GAA is depicted in green sticks near the sites).

**FIGURE 14:**
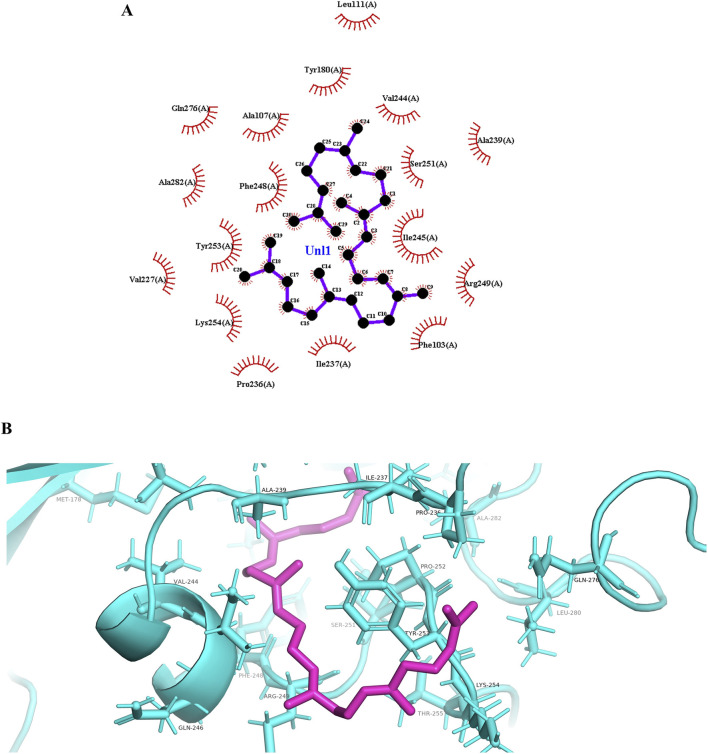
**(A)** LigPlot representations of squalene (Sq) with RDRP (H-bond/electrostatic interactions are shown in green, and the residues that are involved only in hydrophobic interactions are represented by red semicircles). **(B)** Zoomed-in view of the Sq and RDRP (protein ribbons are shown in red, and the bound Sq is depicted in green sticks).

The study used two reference compounds, Sq and GAA, which showed the formation of hydrogen bonds, but the interactions are not as strong as the PPI. In the case of GAA, the binding involves 10 hydrogen bonds, including the residues Arg118 (2.88, 2.93, 4.25, and 4.62 A° ), Phe113 (2.94 and 4.98 A° ), Tyr164 (3.04 A° ), Ile166 (2.96 and 4.83 A° ), and Lys168 (4.94 A° ), with five hydrophobic interactions with Lys116, Met128, Ser129, Ile144, and Tyr167 (Figure 13), while in the case of Sq, none of the poses showed the formation of hydrogen bonds, except hydrophobic interactions with the 17 residues Phe103, Ala107, Leu111, Try180, Val227, Phe236, Ile237, Ala239, Val244, Ile245, Phe248, Arg249, Ser251, Tyr253, Lys254, Gln276, and Ala282 in pose 1 (Figure 14).

### Absorption, distribution, metabolism, excretion, and toxicity analyses

The yolk and white areas are not mutually exclusive. Passive absorption of the drug by the gastrointestinal tract is shown by the white region (high probability), while the yellow region represents brain penetration. Specimens actively effluxed by P-gp (PGP+) are shown in blue, and a non-substrate of P-gp (PGP−) is shown in red. Sq is predicted not to be absorbed and not to be blood–brain barrier (BBB)-permeant as it lies outside the range of the plot (TPSA of 0Åand a WLOGP of 10.21) (Supplementary Figure S5; Supplementary Table S4).

### Simulation dynamic studies

**FIGURE 15:**
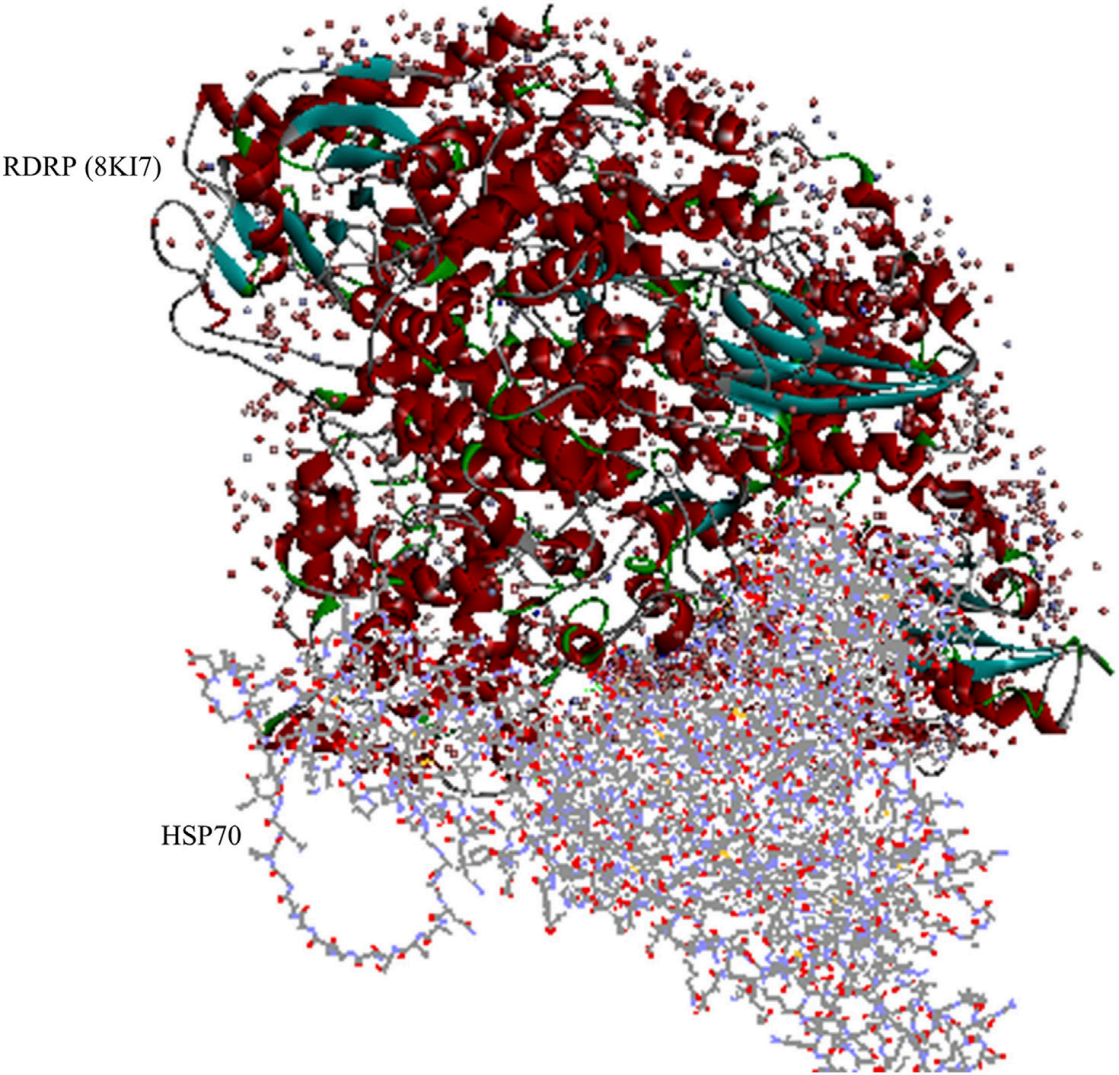
3D interaction mode between RDRP and HSP70.

**FIGURE 16:**
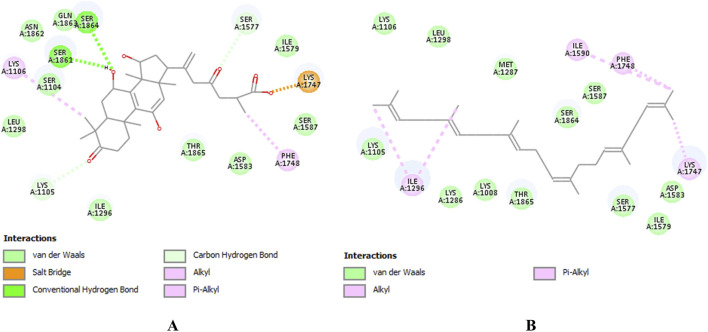
2D representations of interactions of RDRP using DS, GAA **(A)**, and Sq **(B)**. DS, Discovery Studio; GAA, ganoderic acid; Sq, squalene.

MD helps in measuring the stability, compactness, fluctuation, and conformational alterations of the protein complexed with the ligands. The conformations with lower binding energy were selected as starting structures to perform MD simulation. The MD production run for each system was completed for the specified analytical parameters. Furthermore, the Z-docker results of the interaction between the RDRP and HSP70 are shown in Figure 15 (Supplementary Table S5). The binding energies of the RDRP (8KI7) with the reference compounds (GAA and Sq) were found to be RDRP-GAA −42.17 kcal/mol and RDRP-Sq −32.76 kcal/mol, respectively. The RDRP-GAA formed two hydrogen bonds, while Sq has no H-bond formation (Figure 16).

**FIGURE 17:**
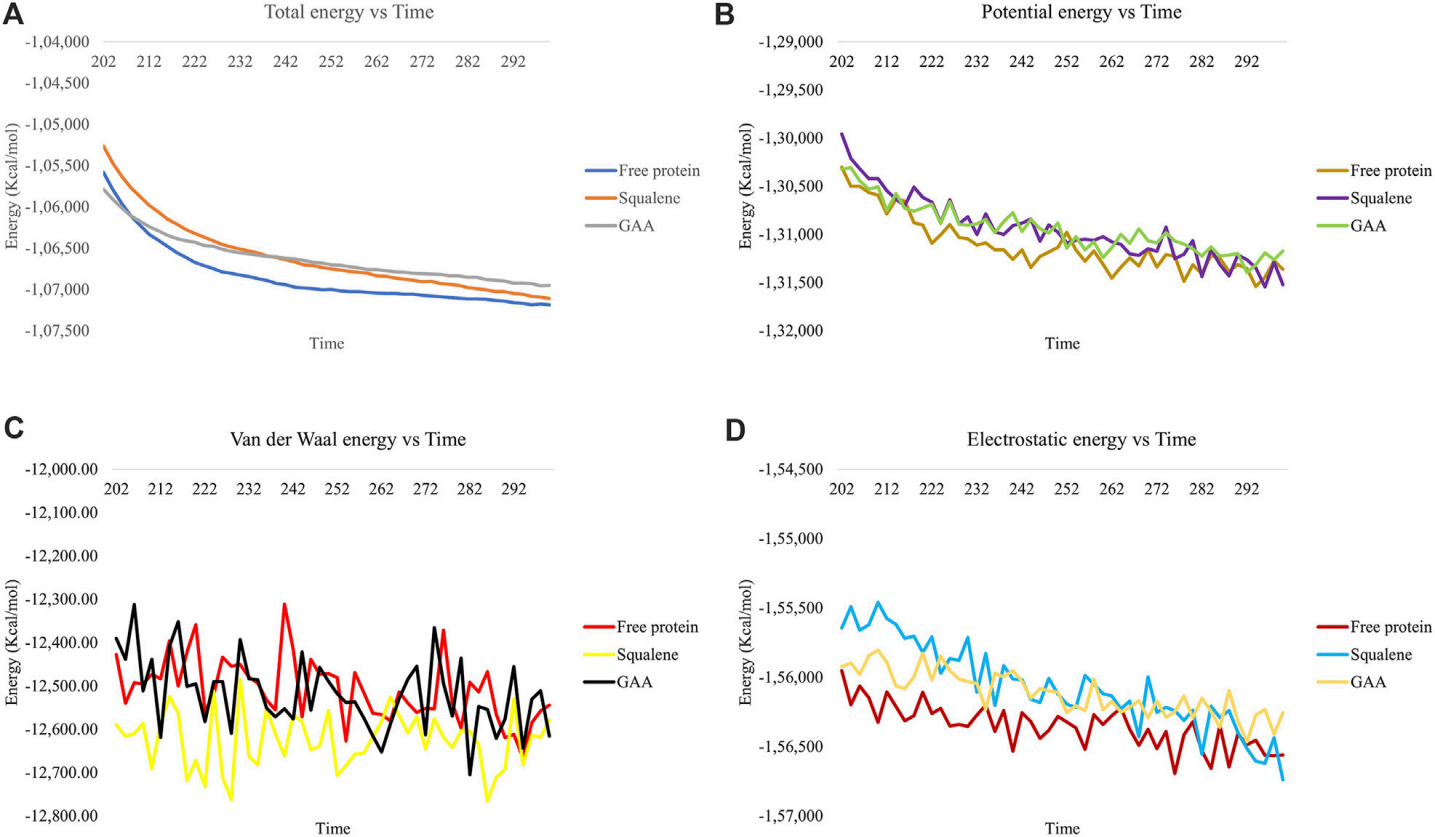
Total, potential, van der Waals, and electrostatic (MMPBSA) energies (Kcal/mol) with respect to time of free protein **(A)**, GAA **(B)**, and Sq **(C)** for the protein 8KI7. GAA, ganoderic acid; Sq, squalene; MMPBSA, molecular mechanics/Poisson–Boltzmann surface area.

The total energy of the free protein (8KI7) showed a gradual decrease with time until 300 ps (Figure 17A). Similarly, the same pattern was observed for Sq and GAA (Figure 17A). The reduction in the total energy of the dynamic simulation studies can confirm the compounds’ stability. Similarly, potential and van der Waal energies also revealed a stable behavior with PL complexes (Figures 17B,C). The MM/PBSA and MM/GBSA binding free energies showed stability of the PL complexes over time, with electrostatic energies (Figure 17D).

### Root mean square deviation and root mean square fluctuations analyses

**FIGURE 18:**
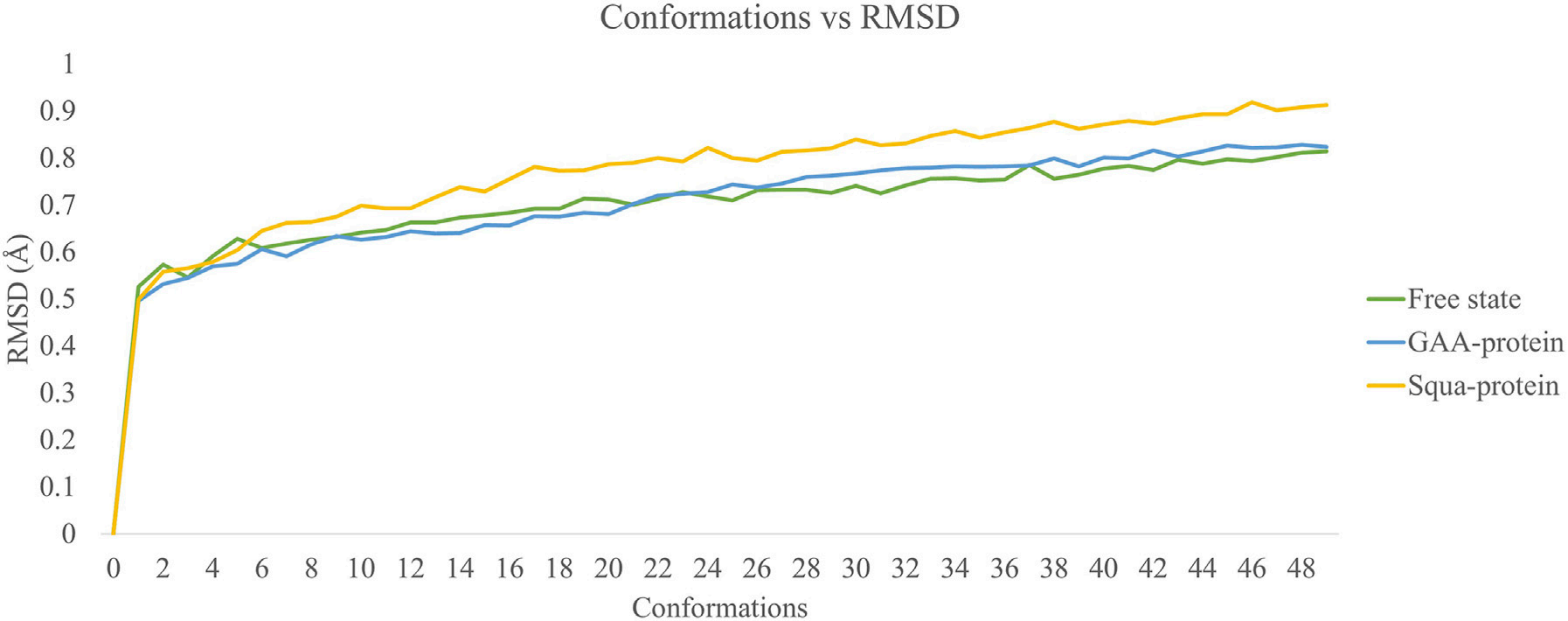
RMSD (in Å) vs. conformations of free protein, GAA, and Sq. GAA, ganoderic acid; Sq, squalene; RMSD, root mean square deviation.

**FIGURE 19:**
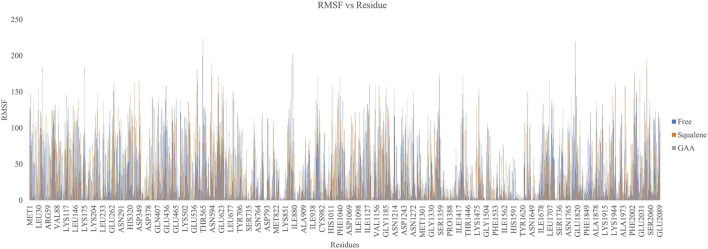
RMSF (in Å) vs. conformations of free protein **(A)**, GAA **(B)**, and Sq **(C)** for the protein 8KI7. GAA, ganoderic acid; Sq, squalene, RMSF, root mean square fluctuations.

**FIGURE 20:**
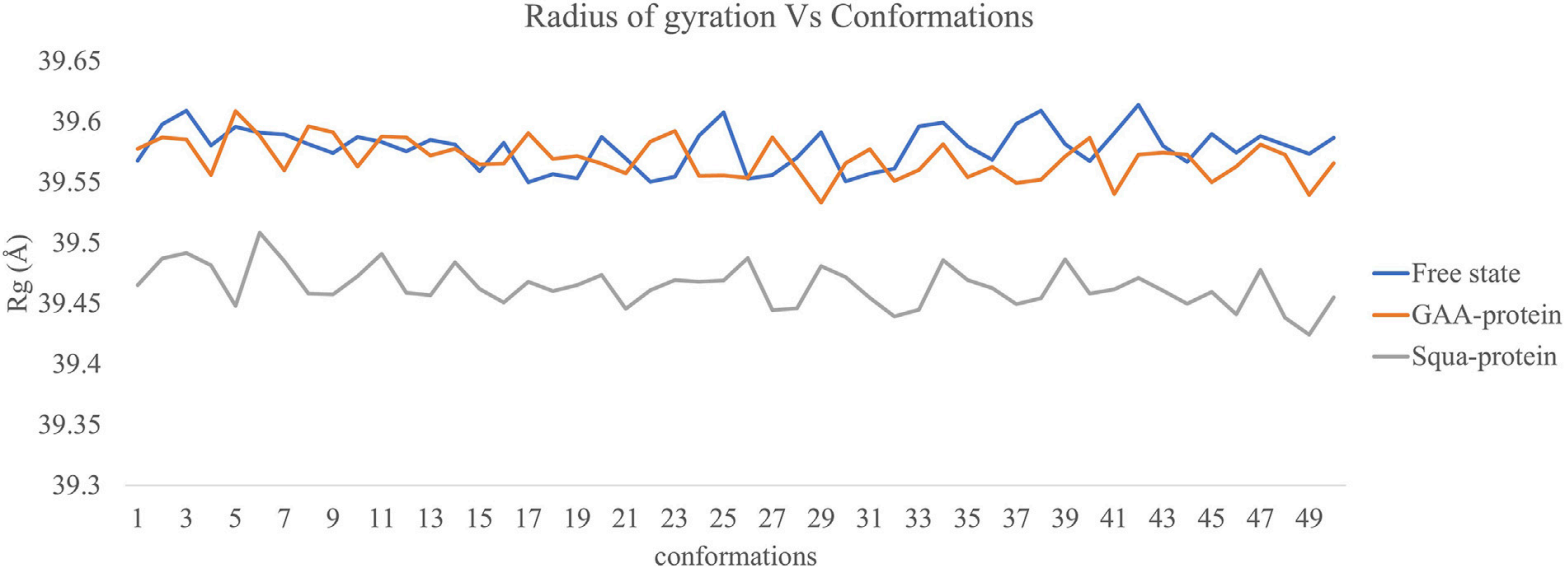
Radius of gyration (in Å) vs. conformations of free protein, GAA, and Sq. GAA, ganoderic acid; Sq, squalene; RMSD, root mean square deviation.

The stability, flexibility, and structural volatility of the receptor–ligand complexes can be described in terms of RMSD and RMSF values and help visualize how the proteins deviate from the initial structure during simulation (Martínez, 2015; Azimi et al., 2021; Ramlal et al., 2024b). The deviations obtained are observed to vary depending on the PL type. For the free protein and GAA docked with the protein (8KI7), the RMSD values ranged from 0.00 to 0.85, while when Sq was docked, it had an RMSD range of 0.00–0.95. In all the cases, there is a steep increase between 1 and 5 conformations, followed by a series of changes in RMSD with each conformation (Figure 18). For the RMSF, all the simulations revealed that the fluctuations over time are stable for each of the ligand–protein complexes (Figure 19). The radius of gyration (Rg) depicts the protein compactness. Here, in this case, the Rg of the systems was found to be between 39.45 and 39.6 Å, respectively. Interestingly, the Rg of the Sq–protein complex was found to be at 39.45 Å, while for the free protein and GAA–protein complex, the Rg ranged from 39.5 to 39.6 Å (Figure 20). These observations indicated that the employed simulation time was enough to obtain equilibrium structures over the simulation. Thus, the structures at the MD equilibrium states were used to investigate the structural specificity of the protein and ligand–protein complexes.

### Post-molecular dynamics simulation computational analyses

**FIGURE 21:**
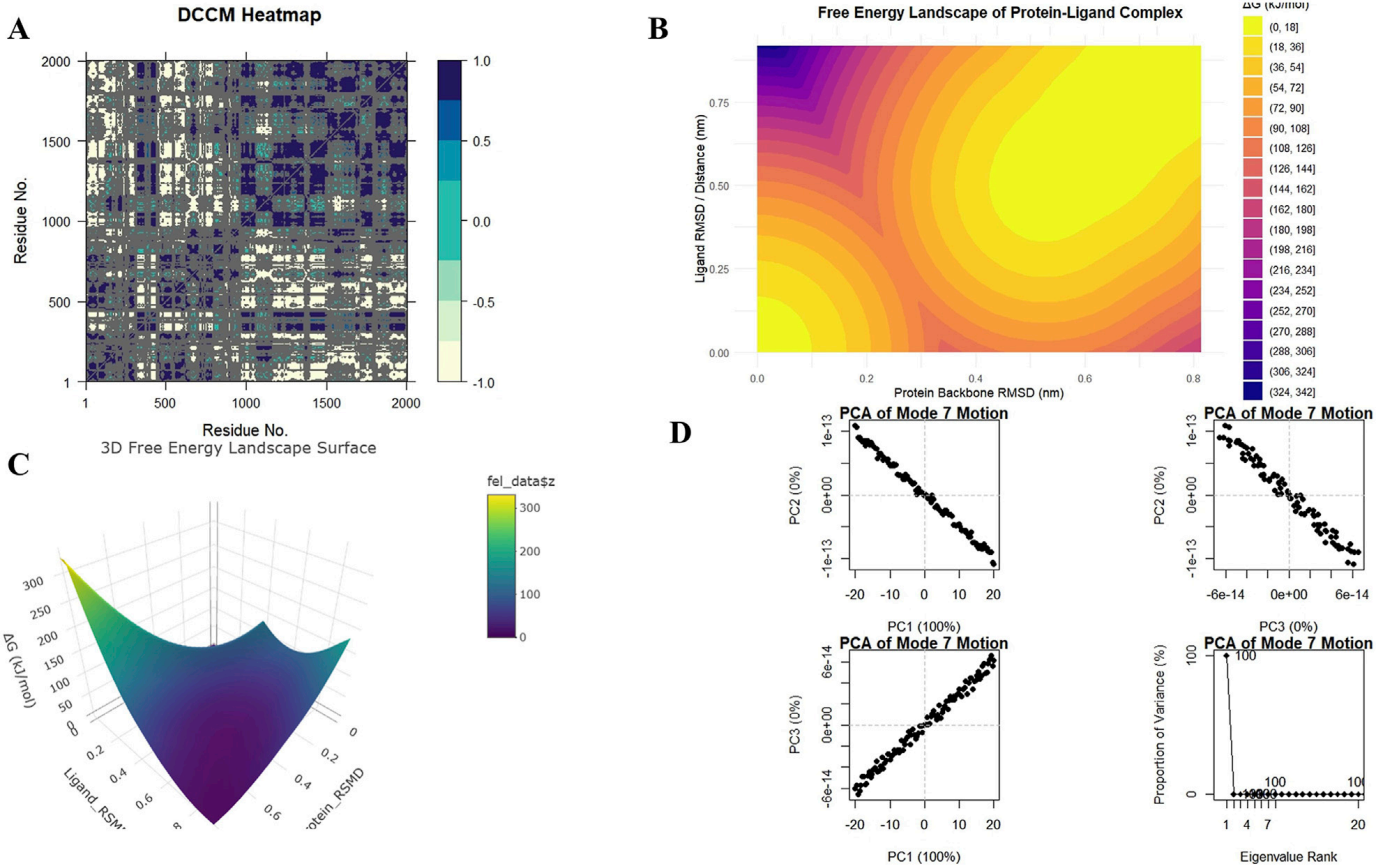
**(A)** Dynamic cross-correlation matrix analysis of the protein–ligand complex showing residue-wise correlated motions for the protein–ligand complex, **(B)** 2D and **(C)** 3D free energy landscape (FEL) plots of the protein–ligand complex, and **(D)** principal component analysis through the normal mode analysis (NMA) during molecular dynamics simulations for the protein (8KI7) and the ligand (squalene; Sq).

**FIGURE 22:**
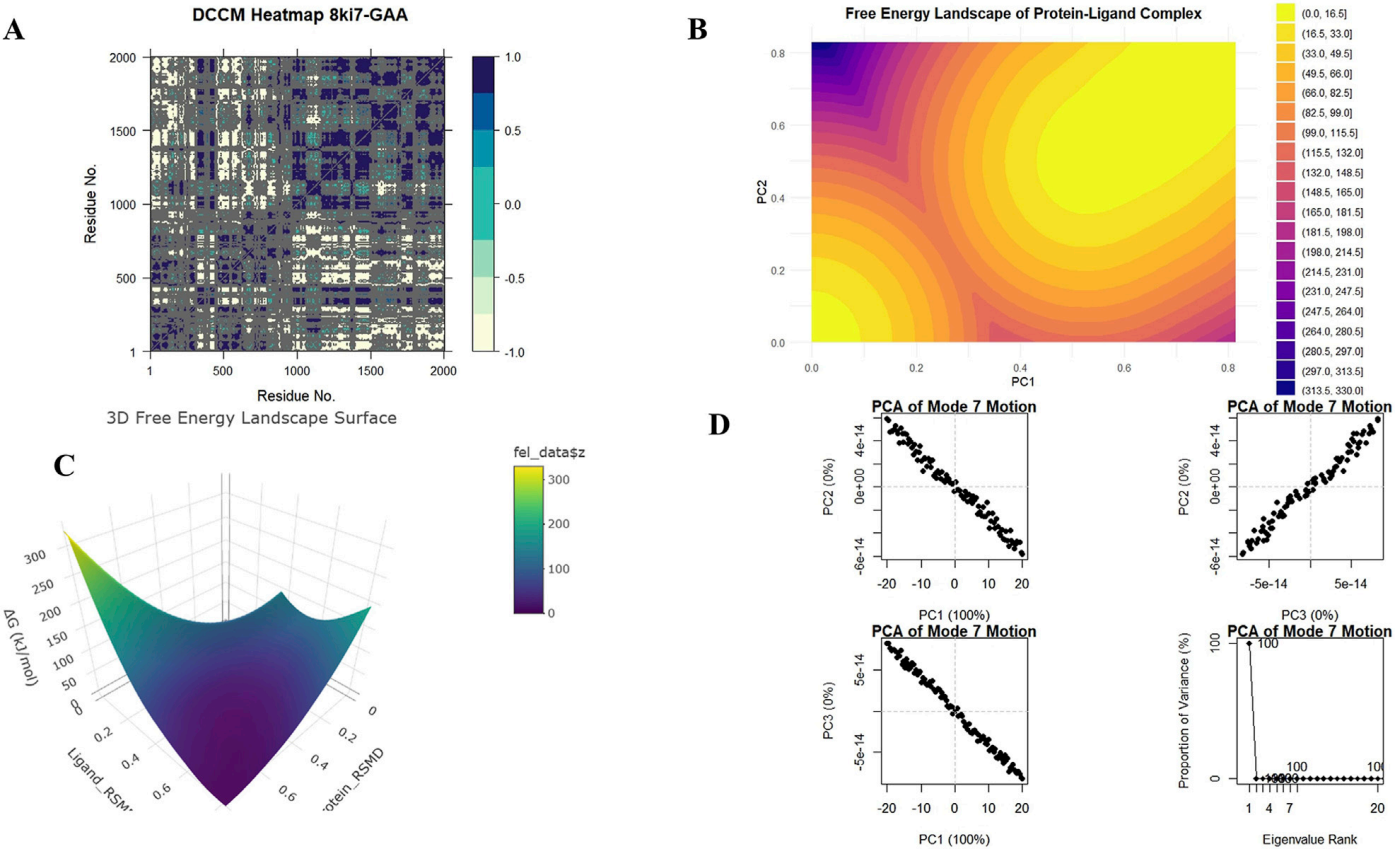
**(A)** Dynamic cross-correlation matrix analysis of the protein–ligand complex showing residue-wise correlated motions for the protein–ligand complex, **(B)** 2D and **(C)** 3D free energy landscape (FEL) plots of the protein–ligand complex, and **(D)** principal component analysis through the normal mode analysis (NMA) during molecular dynamics simulations for the protein (8KI7) and the ligand (ganoderic acid; GAA).

For both the PL complexes, the plots revealed a significant distribution of overall correlated motions (dark regions) and no reduction of anti-correlated motions (red regions). However, they showed the presence of light-colored regions, indicating non-correlation or random motions. The dark regions indicate that the binding pocket is rigid and, at the same time, the ligand has stabilized (Figures 21A, 22A). The thermodynamic stability of the PL complexes is shown in Figures 21B,C, 22B,C, and both represented structurally stable regions. PCA was carried out using the normal mode analysis (Figures 21D, 22D).

## Discussion

*Groundnut bud necrosis virus* (Bunyaviridae) is a tripartite RNA virus containing L, M, and S RNA segments (Jamwal et al., 2025). Studies have shown that GBNV is the causal agent of necrosis in black gram and also affects members of the families Leguminaceae and Solanaceae, including groundnut, tomato, and potato (Divyamani et al., 2024), including soybean. In India, GBNV is known to cause extensive damage, capable of causing significant yield losses (Rai et al., 2020; Divyamani et al., 2024). Therefore, there is an urgent need for developing alternative approaches that could help limit the rate of disease occurrences. This study aimed to identify potential soybean proteins that could be useful against the virus through use of computational methods. PPIs are gaining momentum as they provide insights into binding sites and inhibitory mechanisms (Sangeetha et al., 2021; Srivastava et al., 2024). By using predictive algorithms and computational modeling, insights that could provide strategies to mitigate the virus’s impact on crops, including soybean, can be gained.

This study involves both aspects, namely, sequence and structural analyses of host and viral proteins and molecular docking and dynamics simulations. This integration will provide crucial information about the nature of the proteins, such as evolutionary relationships, active sites, interactive surfaces, and binding pockets. These results can be used for molecular docking for targeting residues and thereby effective development of drugs.

This study reported the physicochemical properties of the proteins, which will be used to develop an understanding of the mechanism of action of both proteins and their inhibition. From secondary structure analysis, it can be observed that both proteins showed more helices than turns and coils. Similarly, post-translational modifications (PTMs) play an important role. They take place in any polypeptide after translation in any cell organelle (nucleus, cytoplasm, endoplasmic reticulum, etc.). They primarily comprise glycosylation—occurring at asparagine, serine (S), and threonine (T) residues—and phosphorylation at S, T, and tyrosine (Y) positions. Additionally, polypeptides may undergo acetylation and methylation. These PTMs help in understanding the functionality of a protein and aid in delineating the evolutionary history and other roles (physicochemical properties, localization, etc.) (Blom et al., 2004). A deeper understanding of RDRP phosphorylation will guide the development of highly effective drugs optimized for stronger target binding (Duflos and Michiels, 2023). The properties of hydrophilicity and hydrophobicity will be very useful for developing drug delivery methods and designing novel therapeutic alternatives. Moreover, the differences in hydrophobicity and hydrophilicity might influence the proteins’ structural stability and lead to differences in their functionality (Ramlal and Samanta, 2022).

Calcium is an important secondary messenger involved in various biotic and abiotic stresses in plants (Guo et al., 2021; Ramlal et al., 2024c; Liu et al., 2025). The presence of various calmodulin-binding sites and motifs in plant proteins indicates several important features and their plausible roles in mitigating abiotic or biotic stresses. Interestingly, these motifs are also found in viral proteins, the functions of which are not yet known. Therefore, they provide a scope for investigation in identifying possible solutions for reducing the infection of the virus in plants. The domains indicate their potential roles in both abiotic- and biotic-mediated stress responses. The encoded IQD proteins contain a plant-specific domain, referred to as the “IQ67 domain,” which is a stretch of 67 conserved amino acid residues. The IQ67 domain contains three copies of the IQ motif (IQxxxRGxxxR) or ([ILV]QxxxRxxxx [R, K]) that are separated by two short sequences with 11 and 15 amino acid residues, respectively. In addition, each IQ motif partially overlaps with three copies of the 1–8–14 motif ([FILVW]×6 [FAILVW]×5 [FILVW]) and four copies of the 1–5–10 motif ([FILVW]×3 [FILV]×4 [FILVW]) (Abel et al., 2005; Levy et al., 2005; Guo et al., 2021; Liu et al., 2025).

In this study, eight soybean proteins were used to investigate the predicted binding affinities with six viral proteins using the HADDOCK score, z-score, and RMSD values, and they were compared with the two reference compounds Sq and GAA (Sangeetha et al., 2021). Several edible fungi produce antiviral compounds, which are being used against different viruses, such as terpenes of *Ganoderma lucidum* against the protease of HIV-1 and AAL lectin of *Agrocybe aegerite* on *Nicotiana glutinosa* L. (Solanaceae) infested with the tobacco mosaic virus (Min et al., 1998; Sun et al., 2003; Ramlal and Samanta, 2022). Recent studies by Sangeetha et al. (2020) and (2021) showed that Sq and GAA are effective against GBNV in cowpeas and found that these organic molecules can be used in the development of formulations against this virus. Similarly, Gayathri et al. (2024) reported the use of endophytic bacteria in controlling GBNV infection in cowpeas and tomato. Therefore, this study utilized them for reference. However, ADMET pharmacokinetic and toxicity properties of the compounds were determined as they are crucial to infer the effectiveness and pharmacological properties of the candidate compound against a target, as well as its safety profile (Daina et al., 2017). The solubility of the compounds in the intestinal fluid, the mode of absorption, penetration of the BBB, plasma protein binding (PPB), and the hepatotoxicity aspects for the phytocompounds are displayed in Supplementary Figure S4 and Supplementary Table S4. Hence, they are not suitable candidate drugs against GBNV. To the best of our knowledge, there are no studies on *in silico* analysis of GBNV and plant-based proteins.

Among the 50 dockings, HSP70 was found to be more potent as compared to the other proteins as it forms more hydrogen bonds and hydrophobic interactions with residues at RDRP sites, ensuring tight binding (Figure 12), which is further correlated with the inhibition constant (Ki = 1.28 $\times 10^{-9}$ M). It was observed that HSP70 is a selective inhibitor for RDRP, while GAA is a moderate inhibitor (Ki = 6.56495 $\times 10^{-5}$ M). In any predicted model, the arrangement of the atoms may be incorrect; otherwise, they are non-randomly distributed due to the geometric and energetic effects within a protein. Therefore, ERRAT validation is necessary to determine the errors in the predicted models. However, the tool is sensitive to structures that are less than 1.5 Å in resolution and non-refined (Wiederstein and Sippl, 2007). Verify3D determines the compatibility of a 3D structure with its skeletal sequence (1D), which, in turn, again confirms the structural quality of a protein (Lüthy et al., 1992).

RDRPs belong to a multi-domain protein class referred to as the structural classification of proteins. It catalyzes the formation of the phosphodiester bonds between ribonucleotides in the presence of a divalent metallic ion on an RNA template (Venkataraman et al., 2018). It is essential in viruses with RNA genomes as it is involved in the processes of replication and transcription (de Farias et al., 2017; Duflos and Michiels, 2023). Therefore, inhibiting the viral RDRP will greatly help in minimizing the risk of viral production and, in turn, disease occurrence. Similarly, plants are continuously exposed to various abiotic and biotic stresses (Ramlal et al., 2023c), and there are several proteins that are employed during abiotic and biotic stresses. One such group of proteins is the HSPs. Different HSPs have been known to be involved during viral infection (ul Haq et al., 2019). For instance, HSP70 is involved in the pathogenesis of the viruses *Tobacco yellow leaf curl virus*, *Cucumber necrosis virus, Red clover necrotic mosaic virus,* and *Tobacco mosaic virus* in tomato and tobacco (Chen et al., 2008; Gorovits et al., 2013; Alam and Rochon, 2016; ul Haq et al., 2019). Therefore, HSP70 could possibly help in mitigating GBNV stress and in crops such as soybean.

The DCCM analysis provided valuable insights into internal residue–residue communication, domain movements, and the influence of ligand binding on protein dynamics. FEL plots were constructed using PCA that was derived from the MD trajectory. The first two principal components (PC1 and PC2), which represented the major collective motions of the system, were selected as reaction coordinates. The Gibbs free energy (ΔG) was calculated using the Boltzmann distribution equation, and the energy landscape was also visualized to identify stable conformational states and transition regions. Lower-energy basins determined energetically favorable and stable conformations of the complex.

## Limitation of the study

The dockings in this study were performed on the proteins selected through homology-based screening due to the lack of availability of viral and host crystal proteins. Hence, this study recommends annotation and crystallization of proteins, and further evaluation in the area is required for proper and complete future studies. Furthermore, this study is confined to only *in silico* analysis, which limits its practical implications. Therefore, experimental evaluation is needed to confirm the PPIs.

## Conclusion and future studies

PPIs play an important role in biological functions. This study presents the first *in silico* analysis of GBNV interactions with soybeans through molecular docking. Among all the docked poses for the determination of the best host–viral PPIs, the results indicated that the soybean HSP70 protein showed potential inhibitory action against the RNA-dependent RNA polymerase of the virus, implying that it could lessen the effects of the virus, thereby reducing the incidence of the disease. However, it is a preliminary work, and therefore, *in vivo* and field trials are required to test the applicability of the inhibitory action of soybean proteins on the virus, including other viruses.

## Data Availability

The original contributions presented in the study are included in the article/Supplementary Material; further inquiries can be directed to the corresponding authors.

## Supplementary material

SUPPLEMENTARY FIGURE S1 Chemical structures of **(A)** ganoderic acid (PubChem: 471002) and **(B)** squalene (PubChem: 638072).

SUPPLEMENTARY FIGURE S2 WebLogo of **(A)** host and **(B)** viral proteins.

SUPPLEMENTARY FIGURE S3 Ramachandran plot of host and virus proteins.

SUPPLEMENTARY FIGURE S4 Graphical representation of z-scores of **(A)** viral and **(B)** host proteins. The black dot represents the z-score of the protein of interest in a plot containing all the z-scores that have been experimentally determined and are currently available in the PDB. X-ray and NMR structures are shown in light- and dark-blue, respectively.

SUPPLEMENTARY FIGURE S5 GAA is predicted as not absorbed and not brain-penetrant (outside the egg). The white region shows a high probability of passive absorption by the gastrointestinal tract, and the yellow region depicts brain penetration.

SUPPLEMENTARY FIGURE S6 Total, potential, van der Waals, and electrostatic (MMPBSA) energies (Kcal/mol) with respect to time of free protein **(A)**, GAA **(B)**, and Sq **(C)** for the protein 4HDH. GAA, ganoderic acid; Sq, squalene; MMPBSA, molecular mechanics/Poisson–Boltzmann surface area.

SUPPLEMENTARY FIGURE S7 RMSF (in Å) vs. conformations of free protein **(A)**, GAA **(B)**, and Sq **(C)** for the protein 8KI7. GAA, ganoderic acid; Sq, squalene, RMSF, root mean square fluctuations.

SUPPLEMENTARY FIGURE S8 (A) Dynamic cross-correlation matrix analysis of the protein–ligand complex showing residue-wise correlated motions for the protein–ligand complex and **(B)** principal component analysis through the normal mode analysis (NMA) during molecular dynamics simulations for the protein (4HDH) and the ligand (squalene; Sq).

SUPPLEMENTARY FIGURE S9 (A) Dynamic cross-correlation matrix analysis of the protein–ligand complex showing residue-wise correlated motions for the protein–ligand complex and **(B)** principal component analysis through the normal mode analysis (NMA) during molecular dynamics simulations for the protein (4HDH) and the ligand (ganoderic acid; GAA).

SUPPLEMENTARY TABLE S1 Energy minimization of host and virus proteins and reference compounds.

SUPPLEMENTARY TABLE S2 Active sites of the proteins as predicted by SPIDDER.

SUPPLEMENTARY TABLE S3 Statistical scores of all the protein-protein interactions performed through HADDOCK.

SUPPLEMENTARY TABLE S4 Toxicity studies of the phytocompounds.

SUPPLEMENTARY TABLE S5 Results of protein–protein docking through Z-docker between RDRP (8KI7) and HSP70 binding clusters, revealing the essential binding amino acids, type of interaction, and size of bonds.
