# Supplementary material for: Molecular docking and dynamics simulation studies uncover the host–pathogen protein–protein interactions in soybean (Glycine max (L.) Merr.) and Groundnut bud necrosis virus: first report
Source: Front Bioinform. 2026 Jul 31;6:1821527. doi: 10.3389/fbinf.2026.1821527 (PMC13473904; doi:10.3389/fbinf.2026.1821527)
Supplement: Supplementary file 1 [file Table2.docx]

**Supplementary Tables**

**Supplementary Table 1**: Energy minimization of host and virus proteins and reference compounds.

| Protein | Energy | Minimised energy |
| --- | --- | --- |
| CAM | -110306.0 kJ/mol | -131451.0 kJ/mol |
| CDK1 | -43033.7 | -56153.0 |
| Cul1 | -376476.2 | -466939.2 |
| GSK1 | -177679.7 | -233097.6 |
| HSP70 | -302608.2 | -376200.6 |
| LOX | -364012.6 | -478934.6 |
| PCNA | -126697.4 | -158864.8 |
| TCP | -53429.3 | -82494.7 |
| CPGB | 287896.7 (score: -1.36) | -409913.4 (score: -0.22) |
| EGPGB | -444050.6 (-4.08) | -648920.6 (-2.83) |
| MPGB | -48044.4 (-1.36) | -68476.8 (-0.17) |
| NPSGB | -27075.2 (-1.96) | -41777.3 (-0.71) |
| NSGB | -32736.1 (-1.99) | -46575.3 (-0.73) |
| RDRPGB | 539603.4 (-3.94) | -816776.6 (-2.54) |
| Squalene* |  |  |
| Ganoderic acid A | 4.106 $\times$ 10^20^ (-0.47) | -122.2 (-1.04) |

*For the Sq, the EM was not returning any results or in other words, the energy was not minimised.

**Supplementary Table 2**: Active sites of the proteins as predicted by SPIDDER.

**Prediction of interacting residues (default -** network majority count >= 5)

| **Protein** | **Interacting residues** |
| --- | --- |
| CAM | M1,S6,E7,E8,I10,V11,D12,K14,E15,A16,G18,L19,F20,K22,D23,T35,V36,I37,R38,S39,L40,D41,Q42,N43,T45,A89,V92,F93,K95,R107,M110,I111,N112,L113,G114,E115,K116,L117,T118,E121,M125,E128,L131,D132,V143,K144,M145,M146,M147,T148,V149,R150  1,6,7,8,10,11,12,14,15,16,18,19,20,22,23,35,36,37,38,39,40,41,42,43,45,89,92,93,95,107,110,111,112,113,114,115,116,117,118,121,125,128,131,132,143,144,145,146,147,148,149,150 |
| CDK1 | M1,Q3,I4,Y6,S7,E8,Y10,F11,D13,Y17,R18,H19,V21,K28,L29,K32,N33,R34,L35,L36,E38,N39,E40,R42,A43,I44,V53,Y55,A56,I57,H58,R59,P60,E61,P62,H63,I64,R68,Q82,Q83,S84,M85,L86,V87,S88,K89  1,3,4,6,7,8,10,11,13,17,18,19,21,28,29,32,33,34,35,36,38,39,40,42,43,44,53,55,56,57,58,59,60,61,62,63,64,68,82,83,84,85,86,87,88,89 |
| Cul1 | M1,E2,K4,I5,S36,E37,Y39,M40,Y43,Y47,T51,R106,R110,H113,D116,R117,S121,R122,G129,G183,L222,N359,V476,L480,R482,L486,E489,R493,D494,S496,H497,I502,D503,T505,T510,T511,I529,K544,H545,R546,L548,T549,G555,T556,H558,P624,K627,V628,N632,A675,V678,R679,K682,S683,R684,Y723,L737,A738  1,2,4,5,36,37,39,40,43,47,51,106,110,113,116,117,121,122,129,183,222,359,476,480,482,486,489,493,494,496,497,502,503,505,510,511,529,544,545,546,548,549,555,556,558,624,627,628,632,675,678,679,682,683,684,723,737,738 |
| GSK1 | M1,T2,S3,V4,G5,V6,A7,P8,T9,S10,G11,L12,R13,E14,A15,S16,G17,H18,G19,A20,A21,G22,V23,D24,R25,L26,E28,E29,V45,E52,T53,I56,N65,E77,R78,V79,V80,G81,H82,G83,S84,F85,G86,V87,V88,K92,T96,L106,Y111,R114,E115,T118,R191,D199,D218,G220,S221,A222,K223,V224,L225,V226,G228,E229,P230,I232,S233,Y234,I235,C236,S237,R238,Y239,R241,I246,T250,T253,T254,P276,E278,  G280,V281,D282,V285,E308,K310,F311,Q313,I314,L345,T348,N370  1,2,3,4,5,6,7,8,9,10,11,12,13,14,15,16,17,18,19,20,21,22,23,24,25,26,28,29,45,52,53,56,65,77,78,79,80,81,82,83,84,85,86,87,88,92,96,106,111,114,115,118,191,199,218,220,221,222,223,224,225,226,228,229,230,232,233,234,235,236,237,238,239,241,246,250,253,254,276,278,280,281,282,285,308,310,311,313,314,345,348,370 |
| HSP70 | M1,A2,D290,S291,D395,L397,L399,E409,T410,A411,G412,G413,V414,T416,V417,L418,P426,I432,Y436,S437,D438,N439,Q440,P441,E451,R452,A453,R454,K456,D457,P470,P471,A472,P473,R474,G475,V476,Q478,A487,A620,G621,P622,G623,G624,D625,V626,P627,M628,G629,A630,D631,M632,P633,A634,A635,G636,A637,G638,P639,K640,I641,E642,E643,V644,D645  1,2,290,291,395,397,399,409,410,411,412,413,414,416,417,418,426,432,436,437,438,439,440,441,451,452,453,454,456,457,470,471,472,473,474,475,476,478,487,620,621,622,623,624,625,626,627,628,629,630,631,632,633,634,635,636,637,638,639,640,641,642,643,644,645 |
| LOX | M1,T2,G3,G4,M5,F6,G7,R8,K9,V33,A38,K39,D40,T41,A42,T43,D44,F45,L46,G47,K48,G49,L50,D51,A52,L53,G54,H55,A56,V57,D58,L60,T61,P100,L300,N301,L302,T410,Q412,Q413,I416,G419,G420,E424,T492,E493  1,2,3,4,5,6,7,8,9,33,38,39,40,41,42,43,44,45,46,47,48,49,50,51,52,53,54,55,56,57,58,60,61,100,300,301,302,410,412,413,416,419,420,424,492,493 |
| PCNA | H44,V45,K110,L126,G127,I128,P129,E130,D174,I175,G176,T177,A178,N179,E232,L233,P234,A252,K254,I255,E256,E257,D258,E259,E260,D261,T262,K263,P264,Q265,V266  44,45,110,126,127,128,129,130,174,175,176,177,178,179,232,233,234,252,254,255,256,257,258,259,260,261,262,263,264,265,266 |
| TCP | T1,Q2,H3,N4,L5,Q6,Q7,G8,L9,D10,S11,G12,D13,N14,P17,T18,L20,V21,R24,E25,R26,M27,K28,L29,K30,R31,T32,Q33,K34,E35,P36,K38,I39,S42,K45,R47,R49,A50,R51,E52,R53,T54,S55,N56,K57,I58,I59,M60,C61,N62,V63,N64,T65,N66,N67,N68,N69,T70,G71,R72,R73,V74,Q75,Q76,Q77,D78,L79,K80,K81,K82,C83,P84,A85,I86,E87,N88,N89,H90,S91,Q92,I93,M94,H95,Q96,S97,S98,S99,S100,P101,T102,H103,N104,L105,V106,G107,N108,E109,T110,P111,R112,D113,D114,F115,N116,V117,I118,E119,E120,S121,I122,V123,I124,K125,R126,K127,L128,K129,P130,S131,L132,M133,S134,S135,S136,H137,H138,H139,H140,H141,H142,H143,Q144,S145,L146,M147,I148,P149,K150,E151,P152,P153,S154,F155  1,2,3,4,5,6,7,8,9,10,11,12,13,14,17,18,20,21,24,25,26,27,28,29,30,31,32,33,34,35,36,38,39,42,45,47,49,50,51,52,53,54,55,56,57,58,59,60,61,62,63,64,65,66,67,68,69,70,71,72,73,74,75,76,77,78,79,80,81,82,83,84,85,86,87,88,89,90,91,92,93,94,95,96,97,98,99,100,101,102,103,104,105,106,107,108,109,110,111,112,113,114,115,116,117,118,119,120,121,122,123,124,125,126,127,128,129,130,131,132,133,134,135,136,137,138,139,140,141,142,143,144,145,146,147,148,149,150,151,152,153,154,155 |
| CPGB | L5,I10,K11,L13,L14,A15,G16,G17,S18,A19,D20,V21,E22,I23,E24,T25,I46,N50,N53,I54,C57,Q60,I61,A63,A64,S67,G68,Y70,V71,F72,C73,G74,I77,M123,Q124,P126,V176,K180,Y189,C193,F206,F214,V218,T228,A229,S231,I232,S233,L234,K235,K236,Y237,E239,Q240,V241,Y243,M244,D245,K246,A247,F248,S249  5,10,11,13,14,15,16,17,18,19,20,21,22,23,24,25,46,50,53,54,57,60,61,63,64,67,68,70,71,72,73,74,77,123,124,126,176,180,189,193,206,214,218,228,229,231,232,233,234,235,236,237,239,240,241,243,244,245,246,247,248,249 |
| EGPGB | N593,L594,P596,G608,K609,Y610,R611,V665,L669,V670,T671,C672,T673,G674,K675,C676,D678,C679,K683,V686,L689,V693,T694,P695,W698,W699,G700,E702,E703,L704,G705,C706,L707,A708,I709,N710,E711,I721,I772,L774,H775,N776,D777,Y778,L801,G802,I816,D817,G818,I819,V823,L827,F832,T833,W834,S835,C836,S837,A838,V839,G840,D841,K843,V859,S873,I906,I907,Q919,C920,A921,L986,T987,V988,D989,I990,Q991,V992,D993,E994,F995,T996,I1003,H1005,F1006  593,594,596,608,609,610,611,665,669,670,671,672,673,674,675,676,678,679,683,686,689,693,694,695,698,699,700,702,703,704,705,706,707,708,709,710,711,721,772,774,775,776,777,778,801,802,816,817,818,819,823,827,832,833,834,835,836,837,838,839,840,841,843,859,873,906,907,919,920,921,986,987,988,989,990,991,992,993,994,995,996,1003,1005,1006 |
| MPGB | T209,P210,I211,A212,A213,E214,N215,T216,T217,C218,D219,V220,V221,P222,I223,N224,R225,K227,S231,A232,L234,I235,E236,A237,C238,K239,L240,M241,I242,P243,K244,G245,I246,K249,Q250,I251,N253,Q254,I255,R264,L267,E268,E269,D272  209,210,211,212,213,214,215,216,217,218,219,220,221,222,223,224,225,227,231,232,234,235,236,237,238,239,240,241,242,243,244,245,246,249,250,251,253,254,255,264,267,268,269,272 |
| NPSGB | 202,203,204,205,206,207,208,209,210,211,212,213,214,215,216,217,218,219,220,221,222,223,224,225,226,227,228,229,230,231,233,234,235,236,237,238,239,241,242,243,244,245,246,247,248,249,250,251,252,253,254,255,256,257,258,259,260,261,262,263,264,265,268,269,270,273,274,277,278,280,281,282,283,284,285,286,287,288,289 |
| NSGB | A8,S9,E10,F11,V12,K13,S14,Y15,G16,T17,R18,D19,N20,R21,A22,I23,N24,D25,C26,Y27,S28,V29,F30,S31,G32,K33,G34,V35,N36,F37,L38,N39,L40,F41,M42,H43,N44,N45,A46,G47,I48,A51,F52,I54,N55,L57,G58,R59,N60,E61,D62,I63,K64,I65,H66,E67,A68,E69,V70,I71,D72,C74,H75,D76,H78,Y79,E81,K82,F83,G84,D86,I87,I88,F89,C90,E91,H92,E93,S95,V97,V98,R99  8,9,10,11,12,13,14,15,16,17,18,19,20,21,22,23,24,25,26,27,28,29,30,31,32,33,34,35,36,37,38,39,40,41,42,43,44,45,46,47,48,51,52,54,55,57,58,59,60,61,62,63,64,65,66,67,68,69,70,71,72,74,75,76,78,79,81,82,83,84,86,87,88,89,90,91,92,93,95,97,98,99 |
| RDRPGB | D90,K91,Y92,K93,E94,L97,M98,F122,M128,I131,F133,C134,S137,T138,N139,I140,I141,N142,Q143,I144,D146,I147,L150,K151,E152,L154,V155,F156,V186,E196,Y199,T200,W203,F206,K207,P215,F216,L217,R219,N230,P231,S232,G233,N234,M235,P236,I237,V238,T240,S242,I245,K254,R259,K260,A263,S264,A265,A266,K267,L268,I269,S270,V271,N272,S273,L274,G275,Q276,L277,I278,F285,R286,R287,Y289,E291,Q293,T294,F295,K296,N297,L299,L300,K302,V303,G304,E305,Y306,M307,N308,R309,S310,S311,E312,V313,F314,F315,S316,H317,N330,Q331,I332,S333,Q334,D335,I336,I337,D338,L339,I340,N341,A342,M343,P344,V348,N349,L353,V354,H355,F356,L357,F358,G359,L362,Y363,K365,E366,T367,L371,F377,G379,Y380,K381,H383,L407,I408,N411,L412,L503,I504,H505,H506,K507,S508,T514,D515,D550,D553,I556,L712,F713,A714,K715,G716,N717,L718,I719,E720,E722,I863,W866,T968,L982,I984,F1060,D1135,G1139,S1140,D1141,Y1143,I1144,R1156,L1159,L1160,M1163,E1164,V1165,E1166,E1169,L1188,M1189,K1190,T1191,N1192,Q1193,D1194,K1195,F1196,Y1197,Y1198,S1200,H1201,P1202,L1203,T1204,V1205,F1238,L1256,G1258,V1261,T1262,F1263,I1264,Q1265,L1266,L1267,T1276,N1277,K1358,L1512,L1513,F1516,N1517,S1590,I1593,L1599,R1600,G1602,S1635,I1636,D1639,I1640,N1641,P1643,L1644,E1662,L1663,S1665,I1666,L1667,Q1674,Y1677,Y1678,K1679,I1680,L1682,D1683,F1684,F1685,N1686,L1687,K1688,F1690,S1691,S1692,L1693,K1694,K1695,V1697,N1698,S1699,L1700,G1701,Y1702,I1703,D1704,L1705,R1706,I1707,F1711,Y1716,I1719,T1720,A1722,D1723,K1724,K1725,M1726,C1728,M1729,V1730,L1732,F1733,S1736,L1737,M1738,S1744,V1747,K1750,F1751,C1780,K1781,L1782,F1783,P1784,R1787,I1793,D1794,A1795,L1796,K1797,P1798,V1799,L1801,E1803,R1804,D1805,L1806,I1809,F1814,L1815,S1828,P1831,E1832,L1835,A1879,L1883,D1884  90,91,92,93,94,97,98,122,128,131,133,134,137,138,139,140,141,142,143,144,146,147,150,151,152,154,155,156,186,196,199,200,203,206,207,215,216,217,219,230,231,232,233,234,235,236,237,238,240,242,245,254,259,260,263,264,265,266,267,268,269,270,271,272,273,274,275,276,277,278,285,286,287,289,291,293,294,295,296,297,299,300,302,303,304,305,306,307,308,309,310,311,312,313,314,315,316,317,330,331,332,333,334,335,336,337,338,339,340,341,342,343,344,348,349,353,354,355,356,357,358,359,362,363,365,366,367,371,377,379,380,381,383,407,408,411,412,503,504,505,506,507,508,514,515,550,553,556,712,713,714,715,716,717,718,719,720,722,863,866,968,982,984,1060,1135,1139,1140,1141,1143,1144,1156,1159,1160,1163,1164,1165,1166,1169,1188,1189,1190,1191,1192,1193,1194,1195,1196,1197,1198,1200,1201,1202,1203,1204,1205,1238,1256,1258,1261,1262,1263,1264,1265,1266,1267,1276,1277,1358,1512,1513,1516,1517,1590,1593,1599,1600,1602,1635,1636,1639,1640,1641,1643,1644,1662,1663,1665,1666,1667,1674,1677,1678,1679,1680,1682,1683,1684,1685,1686,1687,1688,1690,1691,1692,1693,1694,1695,1697,1698,1699,1700,1701,1702,1703,1704,1705,1706,1707,1711,1716,1719,1720,1722,1723,1724,1725,1726,1728,1729,1730,1732,1733,1736,1737,1738,1744,1747,1750,1751,1780,1781,1782,1783,1784,1787,1793,1794,1795,1796,1797,1798,1799,1801,1803,1804,1805,1806,1809,1814,1815,1828,1831,1832,1835,1879,1883,1884 |

**Supplementary Table 3**: Statistical scores of all the protein-protein interactions performed through HADDOCK. (Yellow highlighted ones are selected as the best PPIs from each combination).

| **PPI** | **Cluster size** | **Binding energy (kcal/mol)** | **HADDOCK score** | **RMSD** | **Van der Waals energy** | **Electrostatic energy** | **Desolvation energy** | **Restraints violation energy** | **Z-score** |
| --- | --- | --- | --- | --- | --- | --- | --- | --- | --- |
| **CP_CAM** | 8 | -11.8 | -21.8 $\pm$ 5.8 | 20.5 $\pm$ 0.3 | -64.7 $\pm$ 5.7 | -502.6 $\pm$ 27.8 | 4.5 $\pm$ 2.1 | 1389.8 $\pm$ 44.8 | -1.4 |
| **CP_CDK1** | 7 | -11.0 | -20.4 $\pm$ 14.4 | 9.5 $\pm$ 0.3 | -82.2 $\pm$ 7.5 | -336.7 $\pm$ 48.6 | 5.3 $\pm$ 4.2 | 1239.5 $\pm$ 75.9 | -1.8 |
| **CP_Cul1** | 4 | -13.2 | 94.5 $\pm$ 15.8 | 22.2 $\pm$ 0.1 | -71.5 $\pm$ 7.2 | -306.4 $\pm$ 20.6 | -5.3 $\pm$ 5.3 | 2324.8 $\pm$ 141.5 | -1.5 |
| **CP_GSK3** | 6 | -12.4 | 15.5 $\pm$ 21.2 | 0.6 $\pm$ 0.4 | -88.7 $\pm$ 8.3 | -428.8 $\pm$ 13.7 | -7.2 $\pm$ 3.0 | 1971.1 $\pm$ 216.6 | -1.9 |
| **CP_HSP70** | 8 | -11.1 | 91.8 $\pm$ 26.0 | 20.3 $\pm$ 0.4 | -65.5 $\pm$ 3.8 | -534.8 $\pm$ 70.8 | 26.0 $\pm$ 7.2 | 2383.0 $\pm$ 285.9 | -1.7 |
| **CP_LOX** | 11 | -11.8 | -4.8 $\pm$ 7.9 | 18.3 $\pm$ 0.2 | -66.9 $\pm$ 7.0 | -408.0 $\pm$ 65.0 | 1.2 $\pm$ 2.0 | 1424.7 $\pm$ 146.5 | -2.3 |
| **CP_PCNA** | **23** | **-9.6** | **-40.2** $\boldsymbol{\pm}$ **3.7** | **22.9** $\boldsymbol{\pm}$ **3.7** | **-60.3** $\boldsymbol{\pm}$ **4.5** | **-581.2** $\boldsymbol{\pm}$ **32.8** | **23.8** $\boldsymbol{\pm}$ **3.9** | **1125.1** $\boldsymbol{\pm}$ **35.5** | **-1.8** |
| **CP_TCP** | 8 | -12.4 | 162.6 $\pm$ 11.7 | 27.6 $\pm$ 0.1 | -83.6 $\pm$ 9.1 | -350.3 $\pm$ 18.1 | -6.1 $\pm$ 5.5 | 3224.1 $\pm$ 51.6 | -1.0 |
| **EG_CAM** | 11 | -13.9 | 35.5 $\pm$ 30.6 | 0.8 $\pm$ 0.7 | -76.7 $\pm$ 6.8 | -425.9 $\pm$ 34.0 | 20.1 $\pm$ 4.9 | 1772.6 $\pm$ 364.1 | -1.9 |
| **EG_CDK1** | 9 | -10.7 | 49.9 $\pm$ 9.0 | 9.4 $\pm$ 0.1 | -71.4 $\pm$ 3.5 | -256.1 $\pm$ 39.3 | -8.4 $\pm$ 4.4 | 1809.2 $\pm$ 160.7 | -2.3 |
| **EG_Cul1** | **6** | **-15.7** | **-2.7** | **1.2** $\boldsymbol{\pm}$ **0.7** | **-104.8** $\boldsymbol{\pm}$ **24.8** | **-455.6** $\boldsymbol{\pm}$ **57.0** | **-11.2** $\boldsymbol{\pm}$ **4.7** | **2044.4** $\boldsymbol{\pm}$ **178.3** | **-2.4** |
| **EG_GSK3** | 12 | -11.7 | 113.4 $\pm$ 22.8 | 7.1 $\pm$ 0.1 | -85.9 $\pm$ 10.1 | -253.0 $\pm$ 62.4 | -21.6 $\pm$ 5.2 | 2716.3 $\pm$ 140.4 | -0.9 |
| **EG_HSP70** | 13 | -13.3 | 77.7 $\pm$ 14.3 | 0.8 $\pm$ 0.5 | -90.1 $\pm$ 7.7 | -457.8 $\pm$ 18.6 | 11.7 $\pm$ 3.3 | 2476.7 $\pm$ 129.3 | -1.5 |
| **EG_LOX** | 5 | -15.0 | 90.2 $\pm$ 15.0 | 17.7 $\pm$ 0.0 | -81.6 $\pm$ 3.8 | -290.0 $\pm$ 42.0 | -0.9 $\pm$ 2.6 | 2307.5 $\pm$ 123.5 | -1.4 |
| **EG_PCNA** | 4 | -9.3 | 40.3 $\pm$ 24.1 | 23.5 $\pm$ 0.2 | -86.8 $\pm$ 8.4 | -272.3 $\pm$ 37.1 | 6.0 $\pm$ 5.0 | 1757.0 $\pm$ 193.7 | -1.4 |
| **EG_TCP** | 8 | -16.1 | 191.4 $\pm$ 39.5 | 26.3 $\pm$ 0.1 | -87.7 $\pm$ 13.7 | -392.2 $\pm$ 18.0 | -16.1 $\pm$ 3.8 | 3735.9 $\pm$ 331.2 | 0.0 |
|  |  |  |  |  |  |  |  |  |  |
| **MP_CAM** | 14 | -12.2 | -77.3 $\pm$ 17.4 | 1.3 $\pm$ 0.9 | -62.3 $\pm$ 12.1 | -512.5 $\pm$ 30.1 | 9.0 $\pm$ 5.1 | 784.8 $\pm$ 85.9 | -2.0 |
| **MP_CDK1** | **16** | **-16.1** | **-104.4** $\boldsymbol{\pm}$ **10.0** | **1.1** $\boldsymbol{\pm}$ **0.6** | **-82.2** $\boldsymbol{\pm}$ **9.0** | **-371.6** $\boldsymbol{\pm}$ **14.7** | **3.8** $\boldsymbol{\pm}$ **2.8** | **483.5** $\boldsymbol{\pm}$ **69.3** | **-2.7** |
| **MP_Cul1** | 5 | -10.5 | 72.9 $\pm$ 17.1 | 9.0 $\pm$ 0.1 | -74.4 $\pm$ 7.3 | -287.8 $\pm$ 32.5 | 5.4 $\pm$ 3.2 | 1994.3 $\pm$ 149.2 | -1.4 |
| **MP_GSK3** | 9 | -10.0 | 44.7 $\pm$ 12.0 | 8.7 $\pm$ 0.4 | -58.7 $\pm$ 14.7 | -228.1 $\pm$ 22.2 | -3.7 $\pm$ 3.4 | 1529.1 $\pm$ 112.9 | -1.1 |
| **MP_HSP70** | 4 | -9.6 | 52.9 $\pm$ 33.8 | 11.7 $\pm$ 0.2 | -57.7 $\pm$ 4.7 | -371.8 $\pm$ 29.6 | 13.5 $\pm$ 6.0 | 1714.5 $\pm$ 325.3 | -1.9 |
| **MP_LOX** | 5 | -11.1 | -11.0 $\pm$ 19.8 | 0.7 $\pm$ 0.6 | -61.5 $\pm$ 10.5 | -281.2 $\pm$ 85.5 | -4.4 $\pm$ 6.9 | 1110.8 $\pm$ 148.8 | -2.4 |
| **MP_PCNA** | 13 | -10.2 | -46.5 $\pm$ 9.9 | 7.2 $\pm$ 0.4 | -54.4 $\pm$ 8.0 | -253.9 $\pm$ 52.4 | 2.9 $\pm$ 3.9 | 557.6 $\pm$ 44.7 | -1.5 |
| **MP_TCP** | 11 | -13.4 | 155.0 $\pm$ 20.6 | 1.6 $\pm$ 0.9 | -71.2 $\pm$ 5.8 | -441.0 $\pm$ 64.1 | 16.2 $\pm$ 5.5 | 2982.6 $\pm$ 215.2 | -2.0 |
|  |  |  |  |  |  |  |  |  |  |
| **NPS_CAM** | 7 | -13.2 | -41.7 $\pm$ 17.3 | 13.0 $\pm$ 0.2 | -74.8 $\pm$ 10.9 | -493.7 $\pm$ 59.2 | 12.9 $\pm$ 5.7 | 1189.6 $\pm$ 62.9 | -1.4 |
| **NPS_CDK1** | **13** | **-16.9** | **-120.2** $\boldsymbol{\pm}$ **24.4** | **1.2** $\boldsymbol{\pm}$ **0.9** | **-114.7** $\boldsymbol{\pm}$ **8.9** | **-328.9** $\boldsymbol{\pm}$ **44.9** | **-24.4** $\boldsymbol{\pm}$ **3.2** | **846.1** $\boldsymbol{\pm}$ **119.7** | **-2.0** |
| **NPS_Cul1** | 6 | -13.8 | 44.8 $\pm$ 34.9 | 1.3 $\pm$ 0.7 | -92.2 $\pm$ 3.4 | -360.9 $\pm$ 36.8 | -5.5 $\pm$ 1.8 | 2146.4 $\pm$ 294.2 | -1.8 |
| **NPS_GSK3** | 4 | -17.2 | -5.9 $\pm$ 22.1 | 0.5 $\pm$ 0.3 | -117.1 $\pm$ 3.4 | -440.5 $\pm$ 25.1 | -18.7 $\pm$ 1.3 | 2181.1 $\pm$ 238.1 | -2.0 |
| **NPS_HSP70** | 5 | -17.0 | 99.4 $\pm$ 22.4 | 3.7 $\pm$ 0.8 | -99.0 $\pm$ 18.7 | -269.6 $\pm$ 54.4 | 5.5 $\pm$ 7.5 | 2468.1 $\pm$ 154.7 | -1.3 |
| **NPS_LOX** | 5 | -16.5 | -23.2 $\pm$ 37.3 | 0.7 $\pm$ 0.4 | -95.4 $\pm$ 10.0 | -245.2 $\pm$ 79.5 | -29.8 $\pm$ 4.2 | 1511.5 $\pm$ 266.2 | -2.7 |
| **NPS_PCNA** | 12 | -14.6 | -67.1 $\pm$ 9.1 | 0.6 $\pm$ 0.4 | -93.6 $\pm$ 5.8 | -339.5 $\pm$ 42.5 | 6.6 $\pm$ 3.4 | 878.8 $\pm$ 63.3 | -1.7 |
| **NPS_TCP** | 6 | -13.0 | 184.0 $\pm$ 18.5 | 16.8 $\pm$ 0.1 | -56.5 $\pm$ 5.6 | -474.2 $\pm$ 38.5 | 2.1 $\pm$ 2.6 | 3333.0 $\pm$ 177.7 | -1.3 |
|  |  |  |  |  |  |  |  |  |  |
| **NS_CAM** | **20** | **-13.8** | **-58.9** $\boldsymbol{\pm}$ **9.2** | **20.9** $\boldsymbol{\pm}$ **0.2** | **-92.0** $\boldsymbol{\pm}$ **2.5** | **-427.1** $\boldsymbol{\pm}$ **15.4** | **-8.8** $\boldsymbol{\pm}$ **3.5** | **1273.8** $\boldsymbol{\pm}$ **100.3** | **-2.7** |
| **NS_CDK1** | 11 | -15.7 | -58.1 $\pm$ 8.3 | 18.1 $\pm$ 0.2 | -100.3 $\pm$ 4.6 | -239.7 $\pm$ 32.7 | -26.3 $\pm$ 3.5 | 1164.2 $\pm$ 83.4 | -1.6 |
| **NS_Cul1** | 9 | -13.4 | 28.7 $\pm$ 23.5 | 13.3 $\pm$ 0.0 | -67.9 $\pm$ 12.8 | -610.8 $\pm$ 46.5 | -2.4 $\pm$ 3.5 | 2211.7 $\pm$ 105.3 | -2.1 |
| **NS_GSK3** | 7 | -17.2 | -14.5 $\pm$ 19.4 | 1.5 $\pm$ 0.5 | -110.6 $\pm$ 10.0 | -275.6 $\pm$ 26.5 | -54.8 $\pm$ 3.2 | 2060.2 $\pm$ 101.5 | -2.2 |
| **NS_HSP70** | 6 | -14.4 | 48.3 $\pm$ 24.8 | 3.6 $\pm$ 0.2 | -73.3 $\pm$ 7.8 | -553.4 $\pm$ 66.0 | 3.2 $\pm$ 2.8 | 2290.8 $\pm$ 155.2 | -1.7 |
| **NS_LOX** | 9 | -15.2 | -17.5 $\pm$ 5.4 | 1.6 $\pm$ 0.4 | -93.0 $\pm$ 6.0 | -259.3 $\pm$ 41.4 | -27.4 $\pm$ 1.1 | 1547.2 $\pm$ 92.3 | -1.6 |
| **NS_PCNA** | 11 | -11.7 | -68.7 $\pm$ 6.5 | 1.1 $\pm$ 0.9 | -74.5 $\pm$ 8.3 | -300.4 $\pm$ 39.8 | -17.0 $\pm$ 5.8 | 828.6 $\pm$ 88.4 | -2.1 |
| **NS_TCP** | 5 | -13.0 | 155.0 $\pm$ 9.1 | 2.8 $\pm$ 0.3 | -78.3 $\pm$ 1.2 | -485.7 $\pm$ 22.2 | -7.1 $\pm$ 3.4 | 3123.0 $\pm$ 176.9 | -1.8 |
|  |  |  |  |  |  |  |  |  |  |
| **RD_CAM** | 7 | -17.0 | 59.2 $\pm$ 20.4 | 0.5 $\pm$ 0.3 | -140.8 $\pm$ 11.5 | -359.3 $\pm$ 62.7 | -13.6 $\pm$ 3.9 | 2854.7 $\pm$ 250.0 | -2.4 |
| **RD_CDK1** | 5 | -16.2 | 119.1 $\pm$ 26.2 | 5.9 $\pm$ 0.0 | -95.1 $\pm$ 3.6 | -295.9 $\pm$ 36.8 | -21.1 $\pm$ 3.7 | 2945.1 $\pm$ 235.2 | -1.9 |
| **RD_Cul1** | 25 | -16.1 | 173.3 $\pm$ 39.6 | 1.0 $\pm$ 0.7 | -110.5 $\pm$ 23.9 | -479.2 $\pm$ 53.2 | 11.3 $\pm$ 11.0 | 3684.7 $\pm$ 144.5 | -2.1 |
| **RD_GSK3** | 4 | -13.9 | 518.0 $\pm$ 33.5 | 20.4 $\pm$ 0.2 | -73.5 $\pm$ 10.2 | -180.7 $\pm$ 50.0 | -10.4 $\pm$ 2.6 | 6379.9 $\pm$ 444. | -0.9 |
| **RD_HSP70_I** | **55** | **-19.5** | **90.1** $\boldsymbol{\pm}$ **19.5** | **1.0** $\boldsymbol{\pm}$ **0.6** | **-122.7** $\boldsymbol{\pm}$ **4.9** | **-547.3** $\boldsymbol{\pm}$ **131.0** | **-18.4** $\boldsymbol{\pm}$ **7.5** | **3407.1** $\boldsymbol{\pm}$ **81.3** | **-2.4** |
| **RD_HSP70_refined** | **20** | **-12.12** | **-302.3** $\boldsymbol{\pm}$ **3.1** | **0.7** $\boldsymbol{\pm}$ **0.4** | **- 146.9** $\boldsymbol{\pm}$ **2.7** | **-673.4** $\boldsymbol{\pm}$ **21.6** | **-20.0** $\boldsymbol{\pm}$ **4.0** | **0.0** $\boldsymbol{\pm}$ **0.0** | **0.0** |
| **RD_HSP70_II** | 6 | -12.47 | 315.5 $\pm$ 37.5 | 1.1 $\pm$ 0.7 | -53.1 $\pm$ 16.2 | -745.6 $\pm$ 94.8 | 42.5 $\pm$ 9.6 | 4751.9 $\pm$201.3 | -2.4 |
| **RD_HSP70_III** | 4 | -- | 19.8 $\pm$ 19.4 | 0.7 $\pm$ 0.4 | -104 $\pm$ 7.8 | -555 $\pm$ 25.6 | 26.4 $\pm$ 2.5 | 2083.8 $\pm$ 142.3 | -2.3 |
| **RD_LOX** | 5 | -14.9 | 122.7 $\pm$ 21.7 | 0.6 $\pm$ 0.4 | -125.0 $\pm$ 4.2 | -283.5 $\pm$ 46.0 | 2.9 $\pm$ 4.0 | 3013.7 $\pm$ 215.1 | -2.4 |
| **RD_PCNA** | 5 | -14.5 | 126.0 $\pm$ 21.9 | 16.9 $\pm$ 0.1 | -75.7 $\pm$ 12.0 | -402.8 $\pm$ 44.9 | -4.3 $\pm$ 1.8 | 2865.9 $\pm$ 177.4 | -2.2 |
| **RD_TCP** | 13 | -14.1 | 699.7 $\pm$ 16.8 | 13.4 $\pm$ 0.1 | -85.1 $\pm$ 5.9 | -239.4 $\pm$ 23.9 | -43.6 $\pm$ 3.8 | 8762.7 $\pm$ 299.0 | -1.4 |
